# Supplementary material for: Cerebrospinal fluid oligoclonal bands in Neuroborreliosis are specific for Borrelia burgdorferi
Source: PLoS One. 2020 Sep 25;15(9):e0239453. doi: 10.1371/journal.pone.0239453 (PMC7518929; doi:10.1371/journal.pone.0239453)
Supplement: S1 Table — Abbreviations: NCM: nitrocellulose membrane. NB: neuroborreliosis. OCB: oligoclonal bands. SD: standard deviation. (PDF) [file pone.0239453.s001.pdf]

Run	Visual analysis		Photometric analysis	
	**No. of OCB uncoated NCM**	**No. of OCB pre-coated NCM**	**No. of OCB uncoated NCM**	**No of. OCB pre-coated NCM**
1	19	7	9	6
2	19	6	9	7
3	13	13	13	12
4	10	10	13	12
5	10	12	13	11
6	15	9	13	9
Mean±SD	14±4	10±3	12±2	10±3
Inter-assay precision	28%	29%	14%	27%
