# Supplementary material for: Cerebrospinal fluid oligoclonal bands in Neuroborreliosis are specific for Borrelia burgdorferi
Source: PLoS One. 2020 Sep 25;15(9):e0239453. doi: 10.1371/journal.pone.0239453 (PMC7518929; doi:10.1371/journal.pone.0239453)
Supplement: S2 Table — Abbreviations: NCM: nitrocellulose membrane. OCB: oligoclonal bands. SD: standard deviation. (PDF) [file pone.0239453.s002.pdf]

| Run | Visual analysis | | Photometric analysis | |
|---|---|---|---|---|
| | No. of OCB uncoated NCM | No. of OCB pre-coated NCM | No. of OCB uncoated NCM | No of. OCB pre-coated NCM |
| 1 | 9 | 0 | 9 | 0 |
| 2 | 13 | 0 | 13 | 0 |
| 3 | 9 | 0 | 9 | 0 |
| 4 | 10 | 0 | 10 | 0 |
| 5 | 9 | 0 | 7 | 0 |
| 6 | 9 | 0 | 7 | 0 |
| **Mean±SD** | 10±2 | 0 | 9±2 | 0 |
| **Inter-assay precision** | 16% | 0% | 24% | 0% |
