# Supplementary figures and images for: Cerebrospinal fluid oligoclonal bands in Neuroborreliosis are specific for Borrelia burgdorferi
Source: PLoS One. 2020 Sep 25;15(9):e0239453. doi: 10.1371/journal.pone.0239453 (PMC7518929; doi:10.1371/journal.pone.0239453)

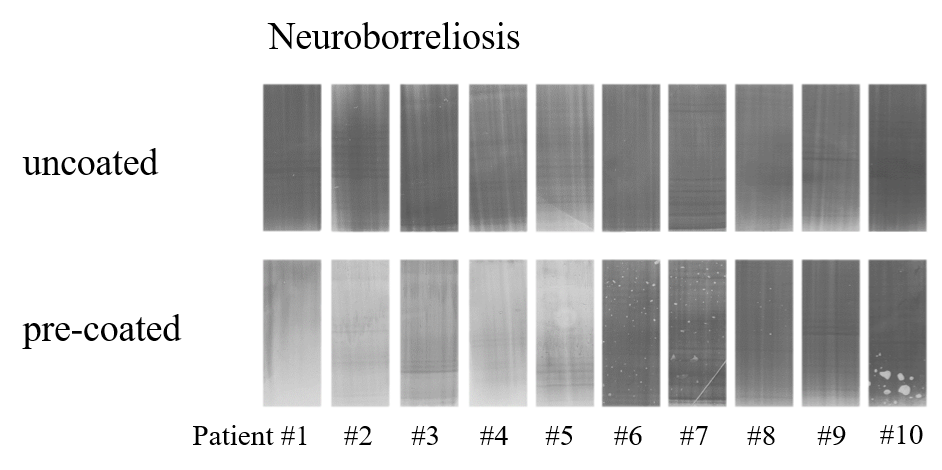

Supplement: S1 Fig — (TIFF) [file pone.0239453.s003.tiff]

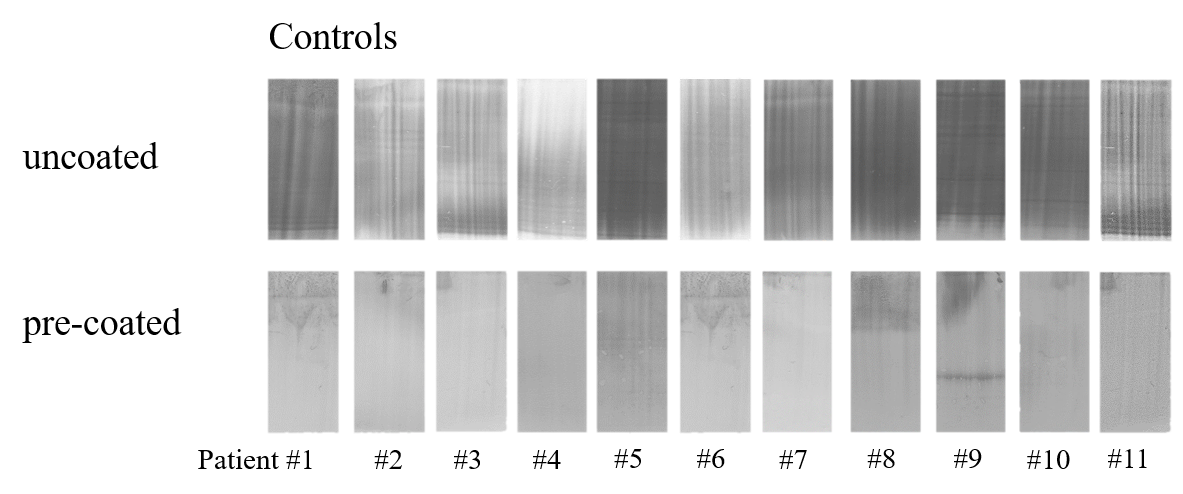

Supplement: S2 Fig — (TIFF) [file pone.0239453.s004.tiff]
