# Supplementary material for: Cerebrospinal fluid oligoclonal bands in Neuroborreliosis are specific for Borrelia burgdorferi
Source: PLoS One. 2020 Sep 25;15(9):e0239453. doi: 10.1371/journal.pone.0239453 (PMC7518929; doi:10.1371/journal.pone.0239453)
Supplement: S1 Raw image — (PDF) [file pone.0239453.s005.pdf]

NB patient #1 uncoated

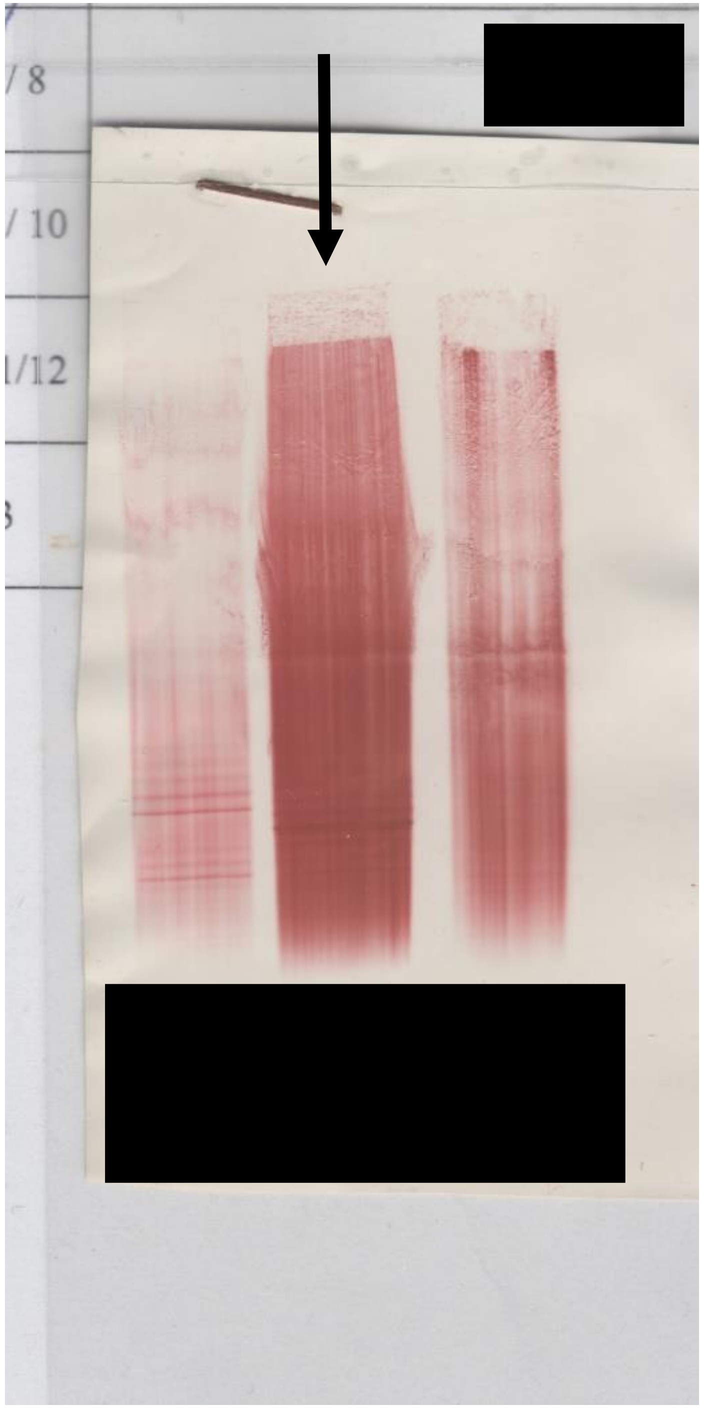

NB patient #1 precoated

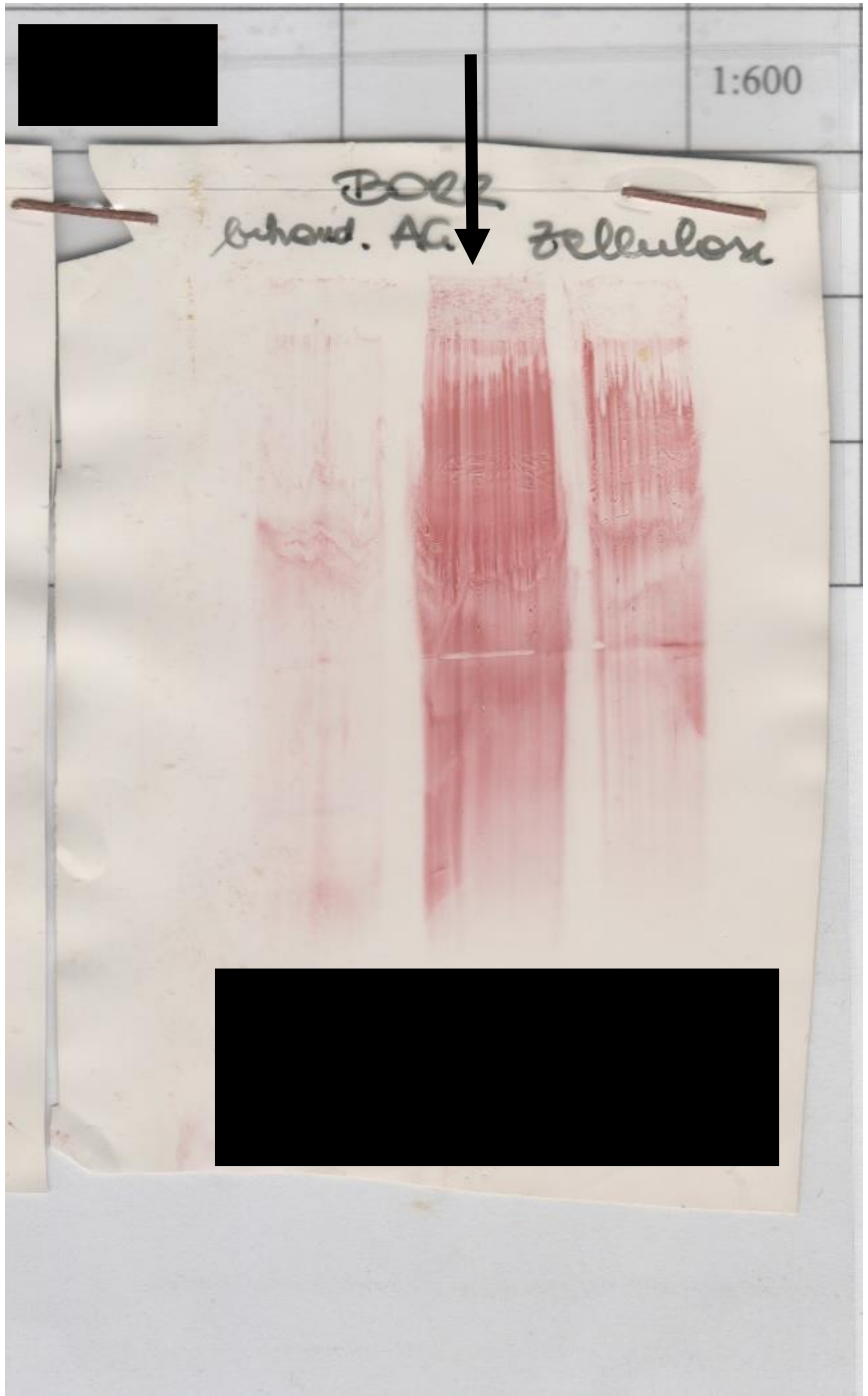

NB patient #2 uncoated

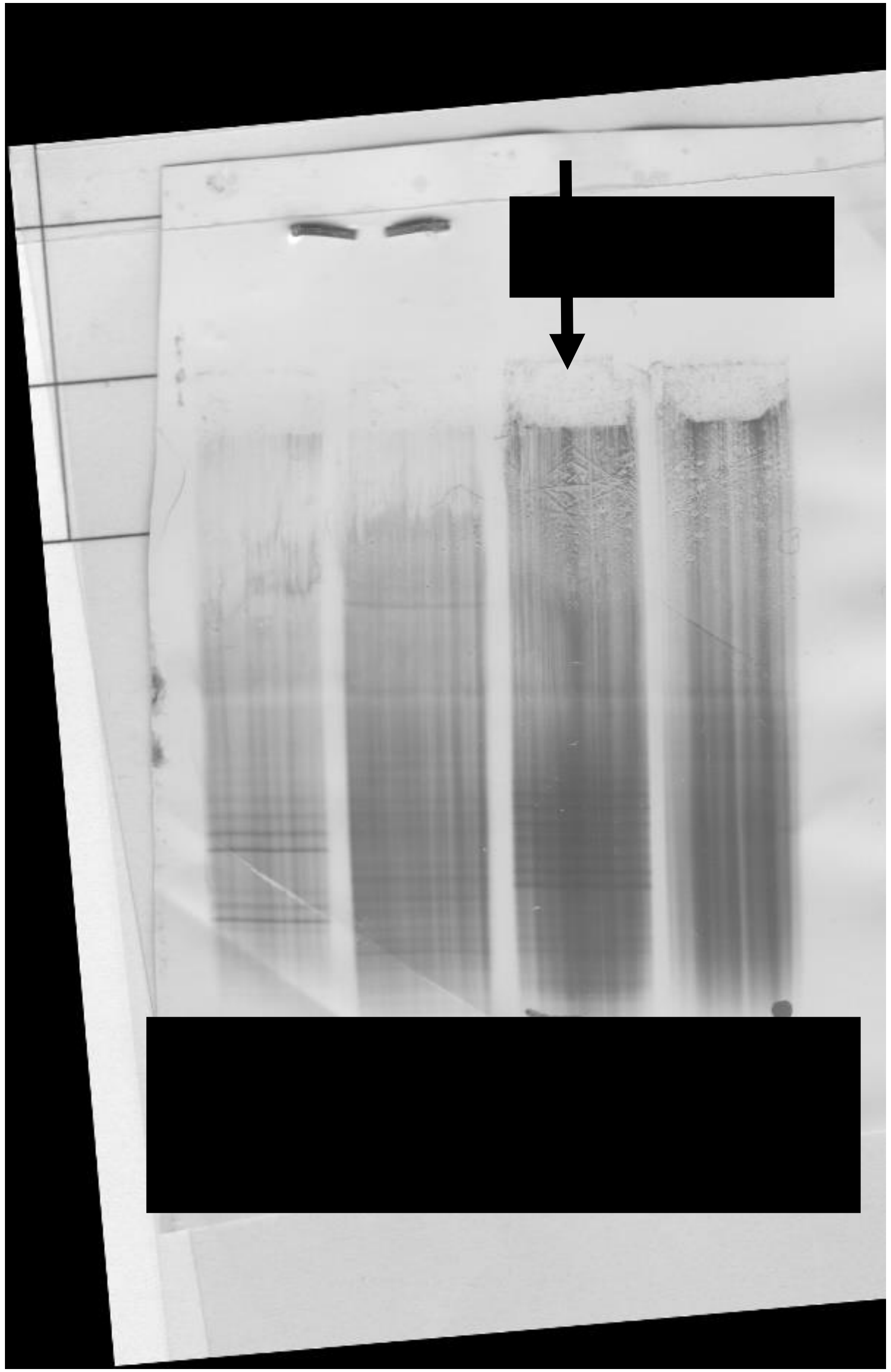

NB patient #2 precoated

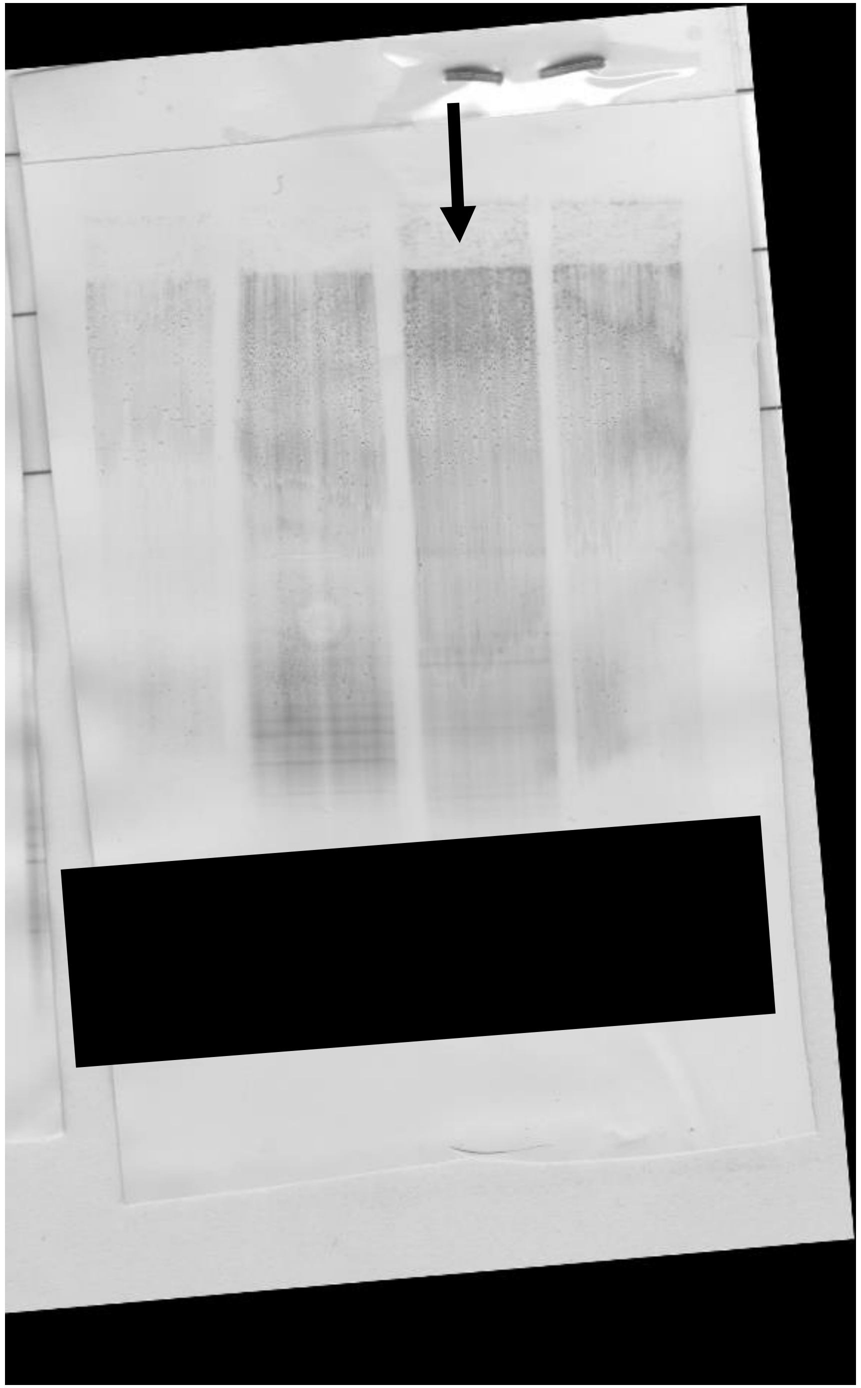

[redacted]

NB patient #3 uncoated

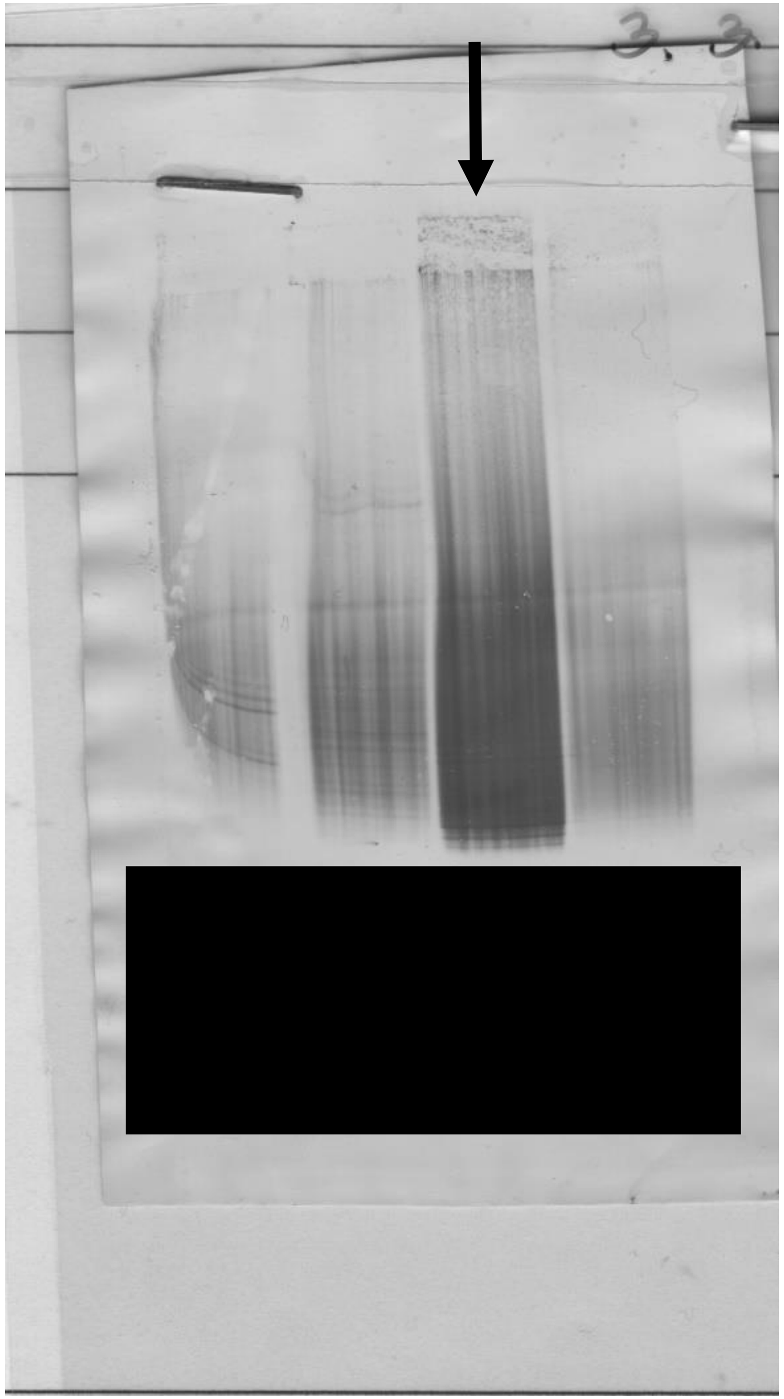

NB patient #3 precoated

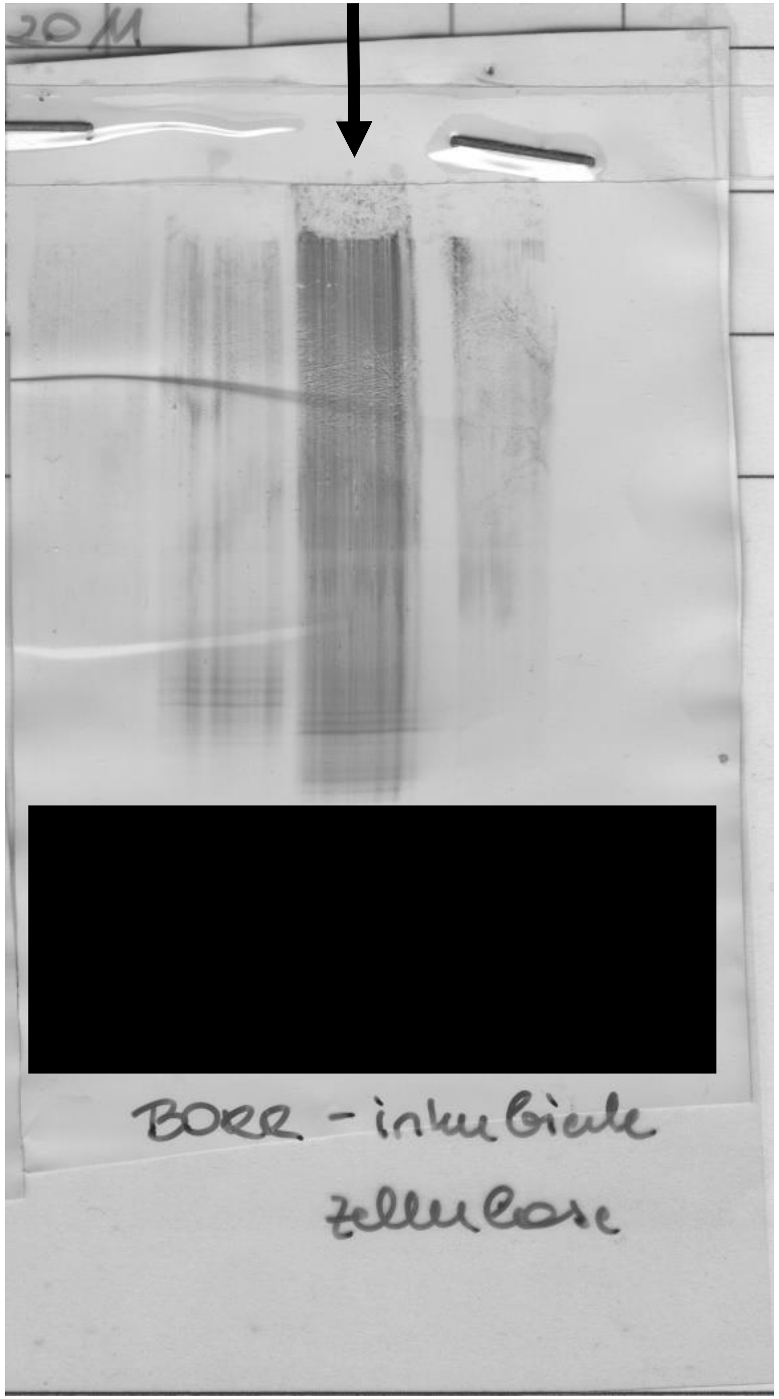

NB patient #4 uncoated

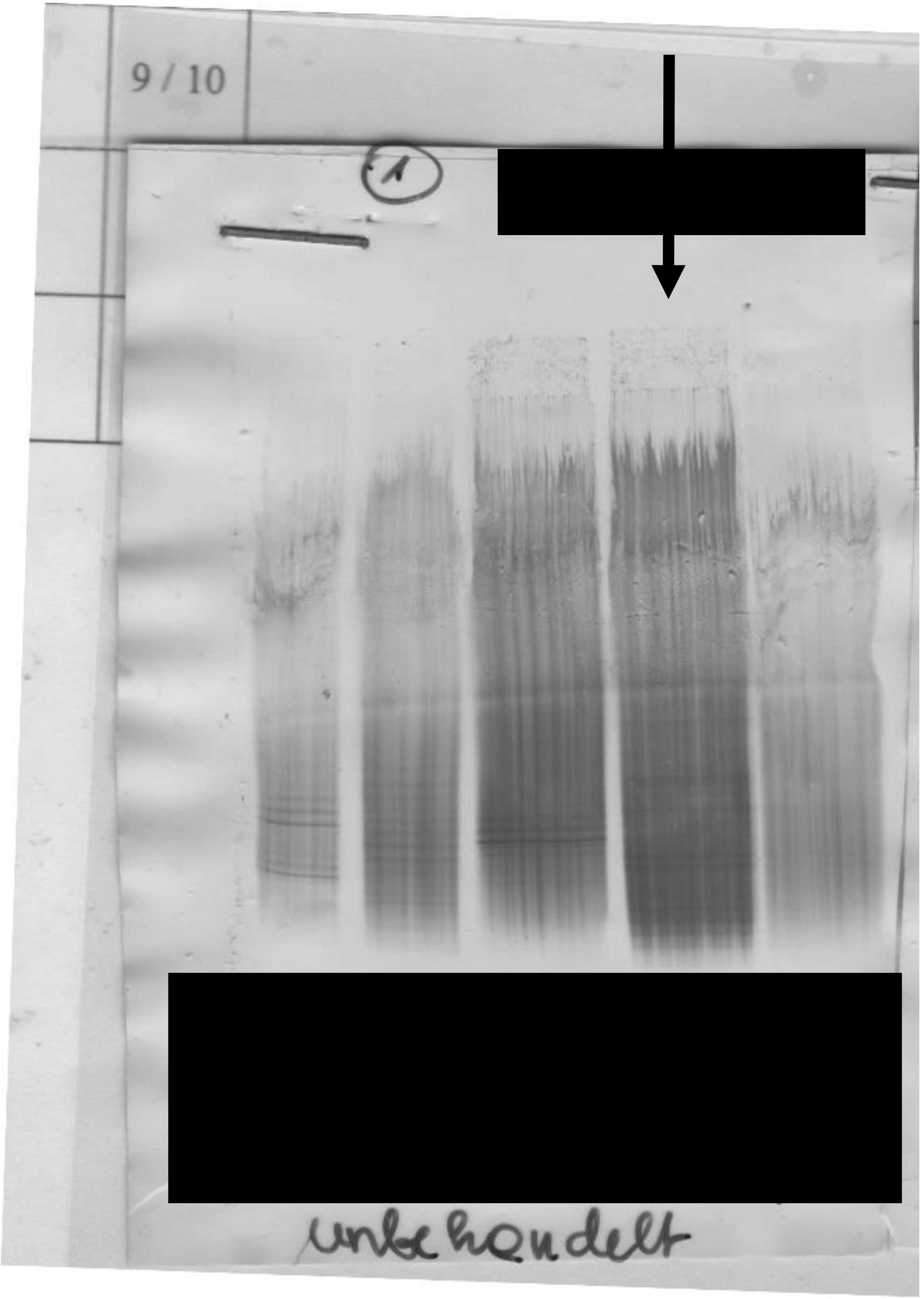

NB patient #4 precoated

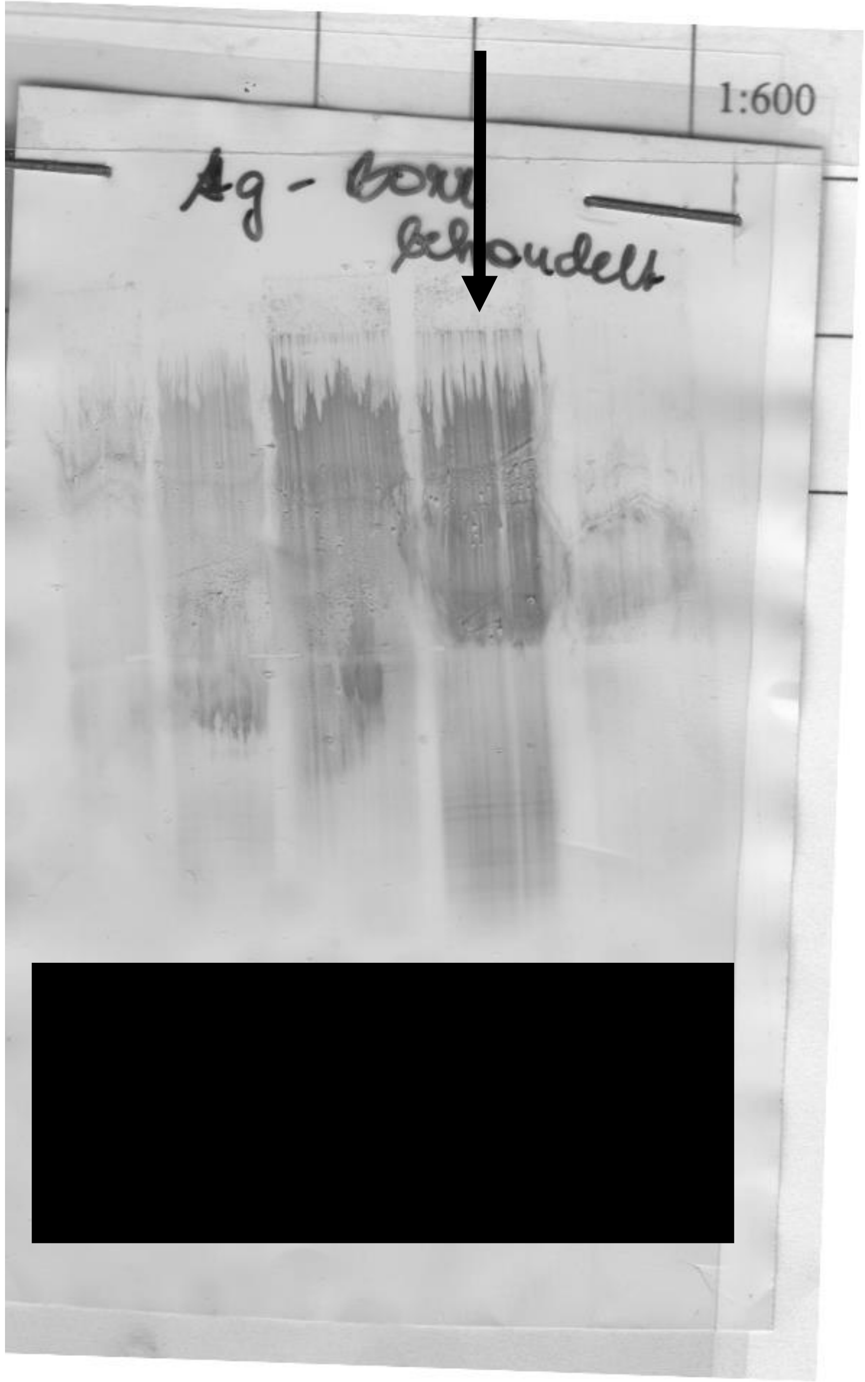

NB patient #5 uncoated

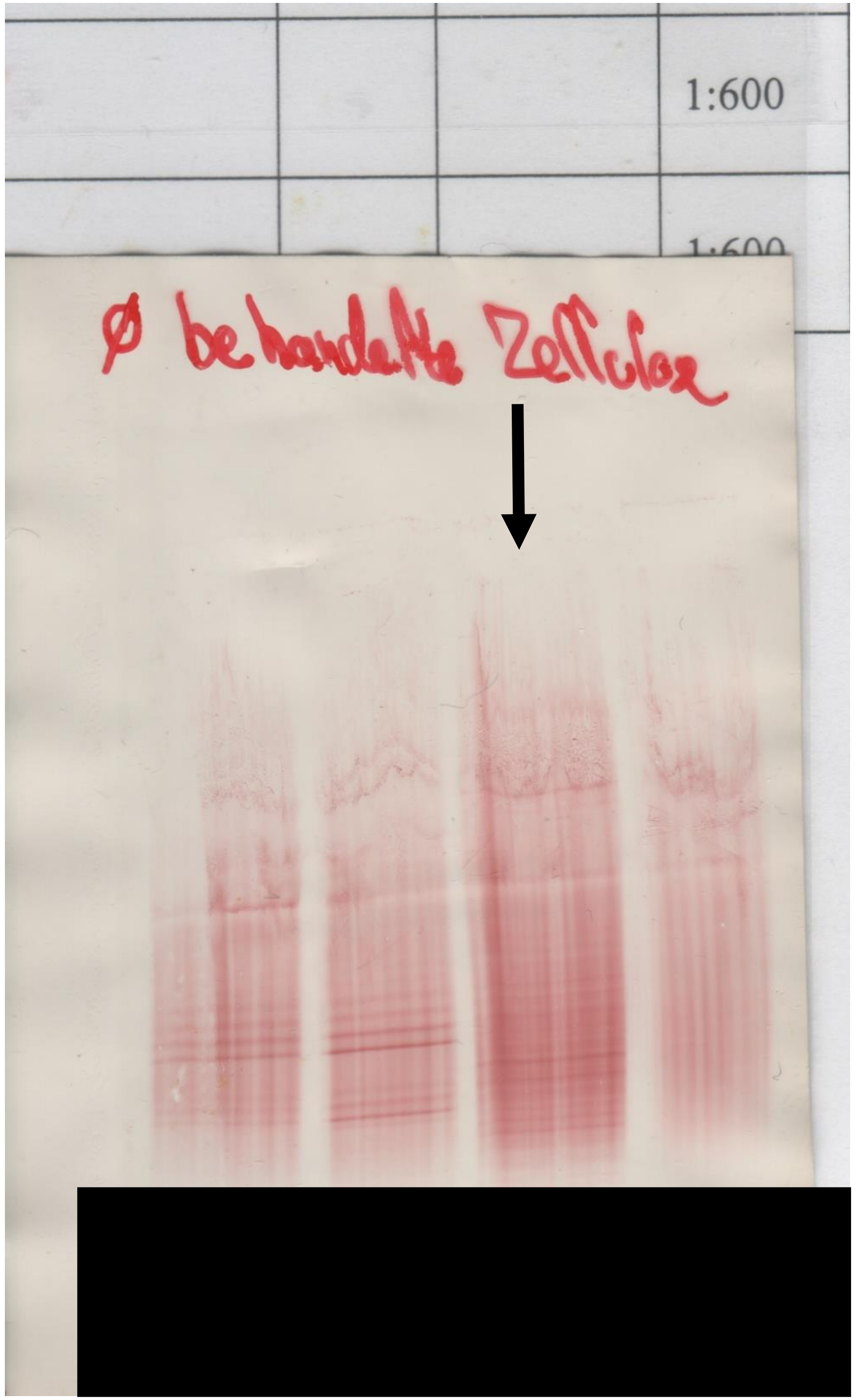

[redacted]

NB patient #5 precoated

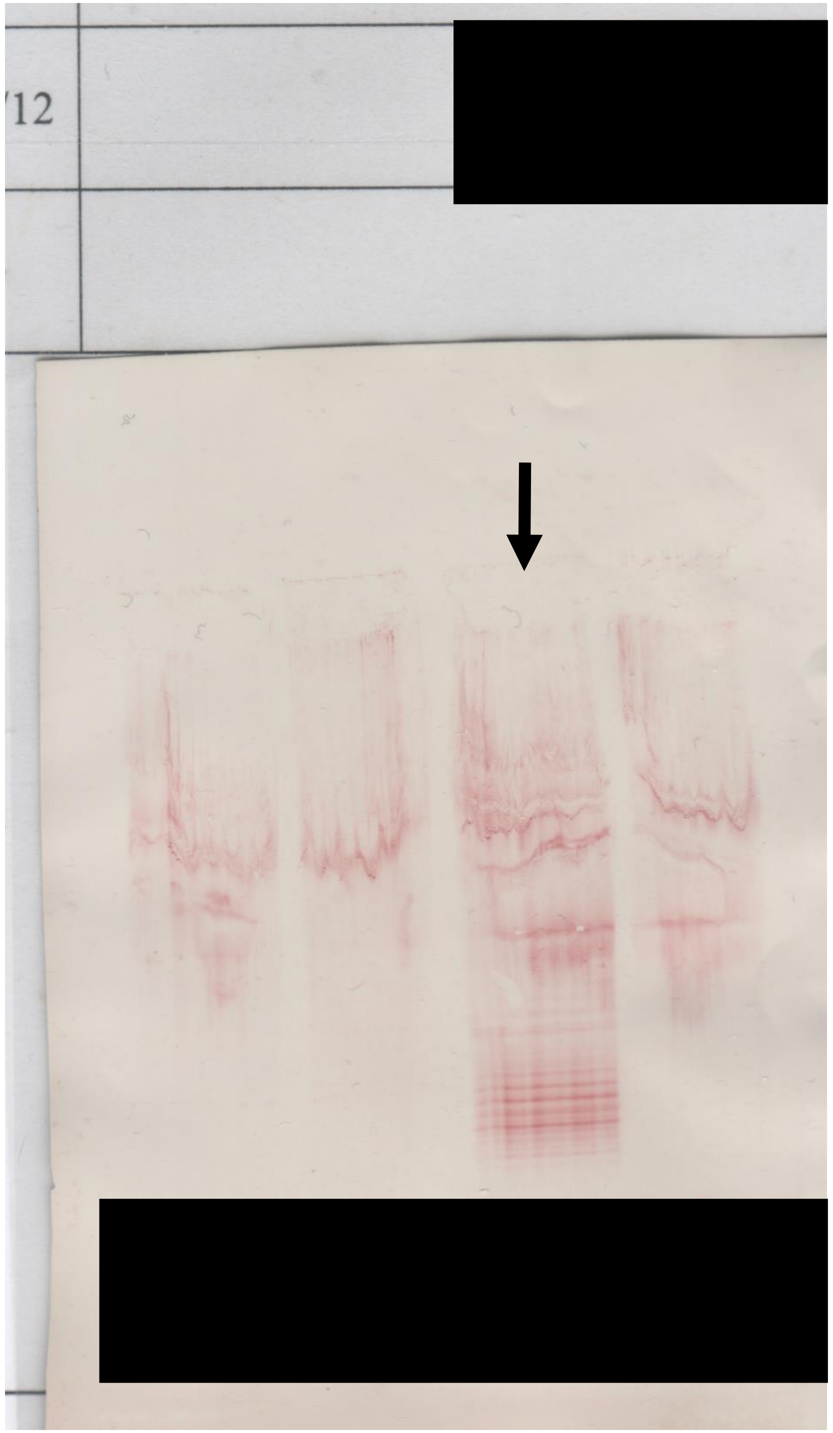

NB patient #6 uncoated

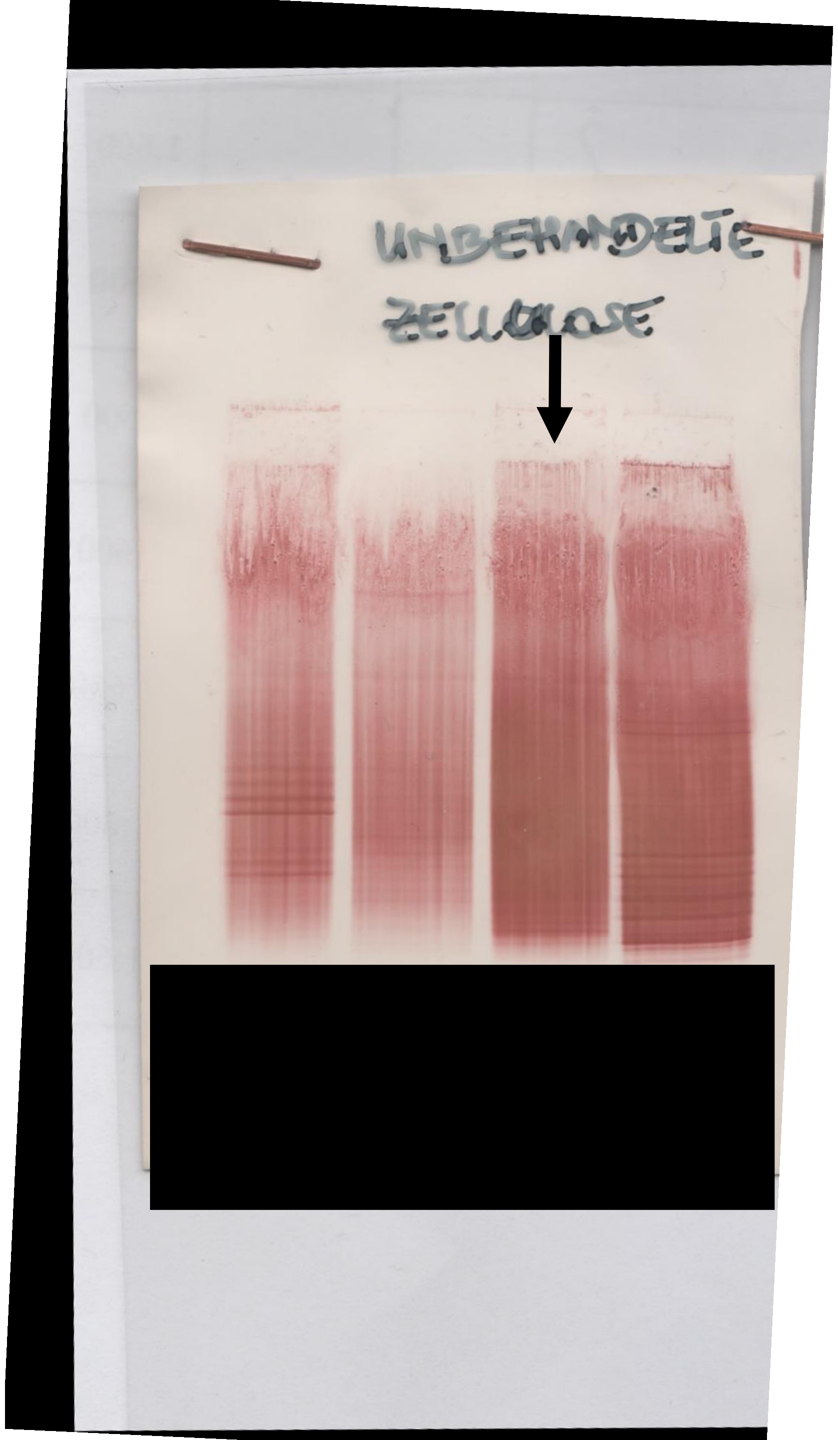

[redacted]

NB patient #6 precoated

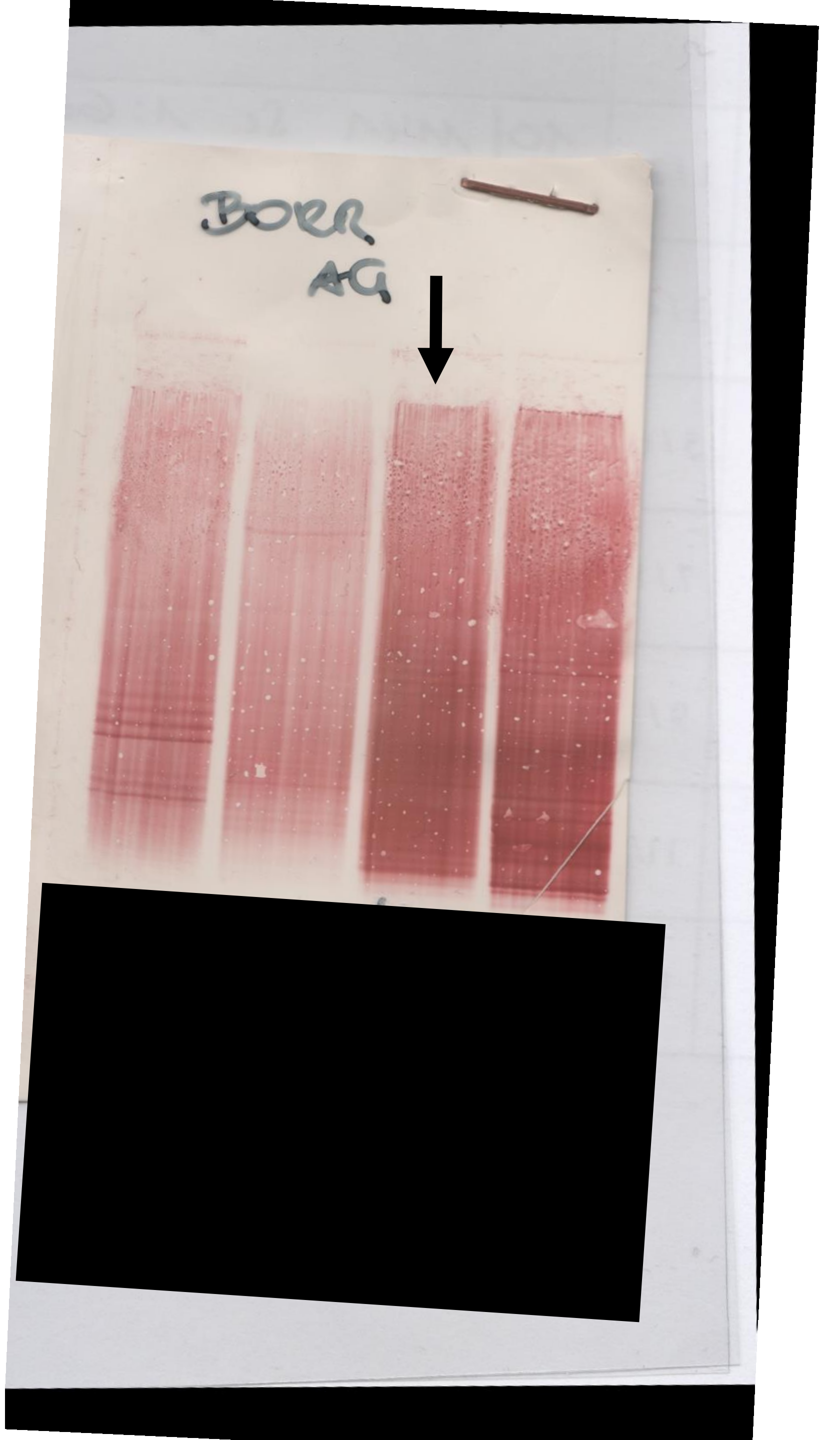

[redacted]

NB patient #7 uncoated

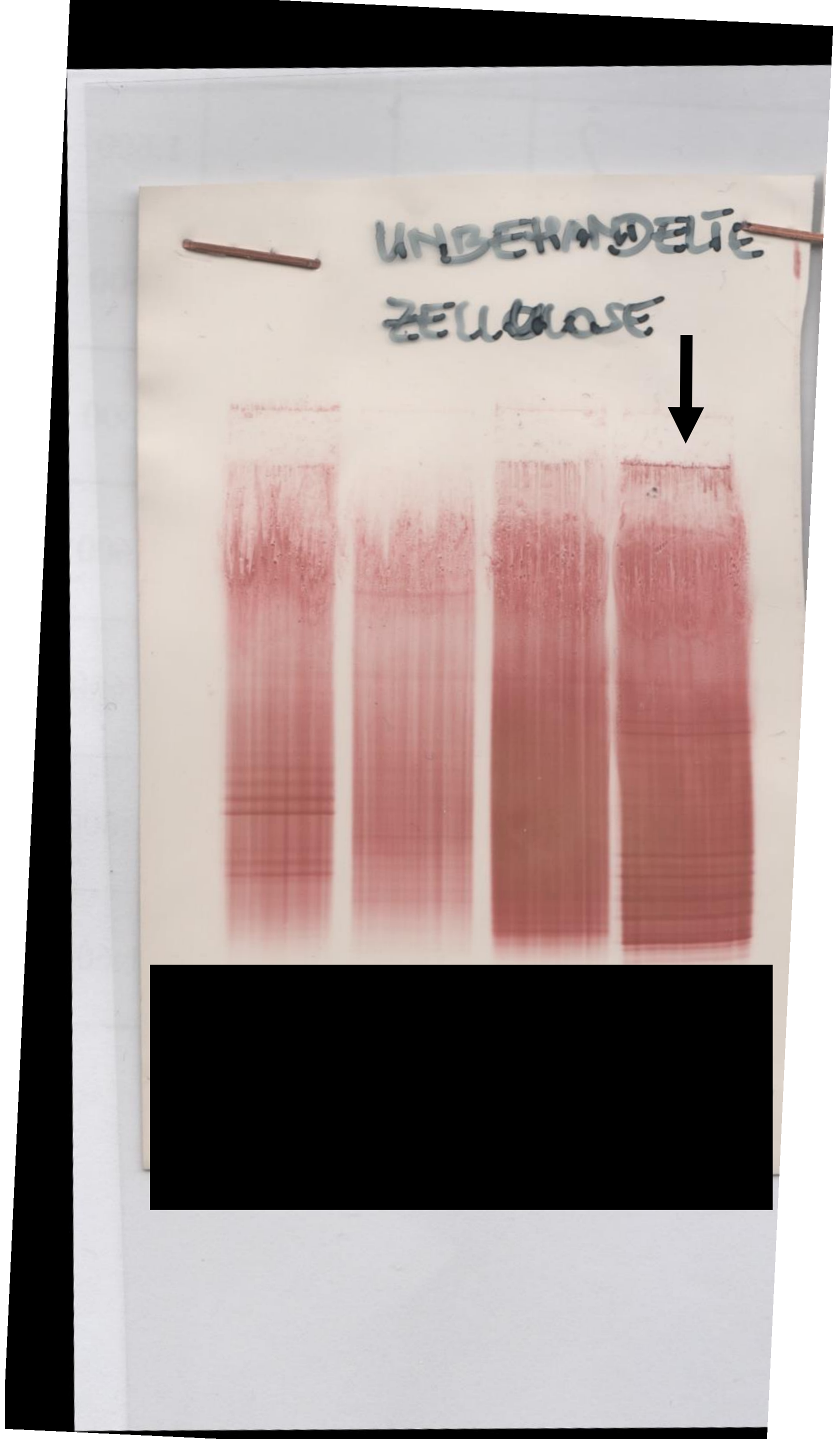

[redacted]

NB patient #7 precoated

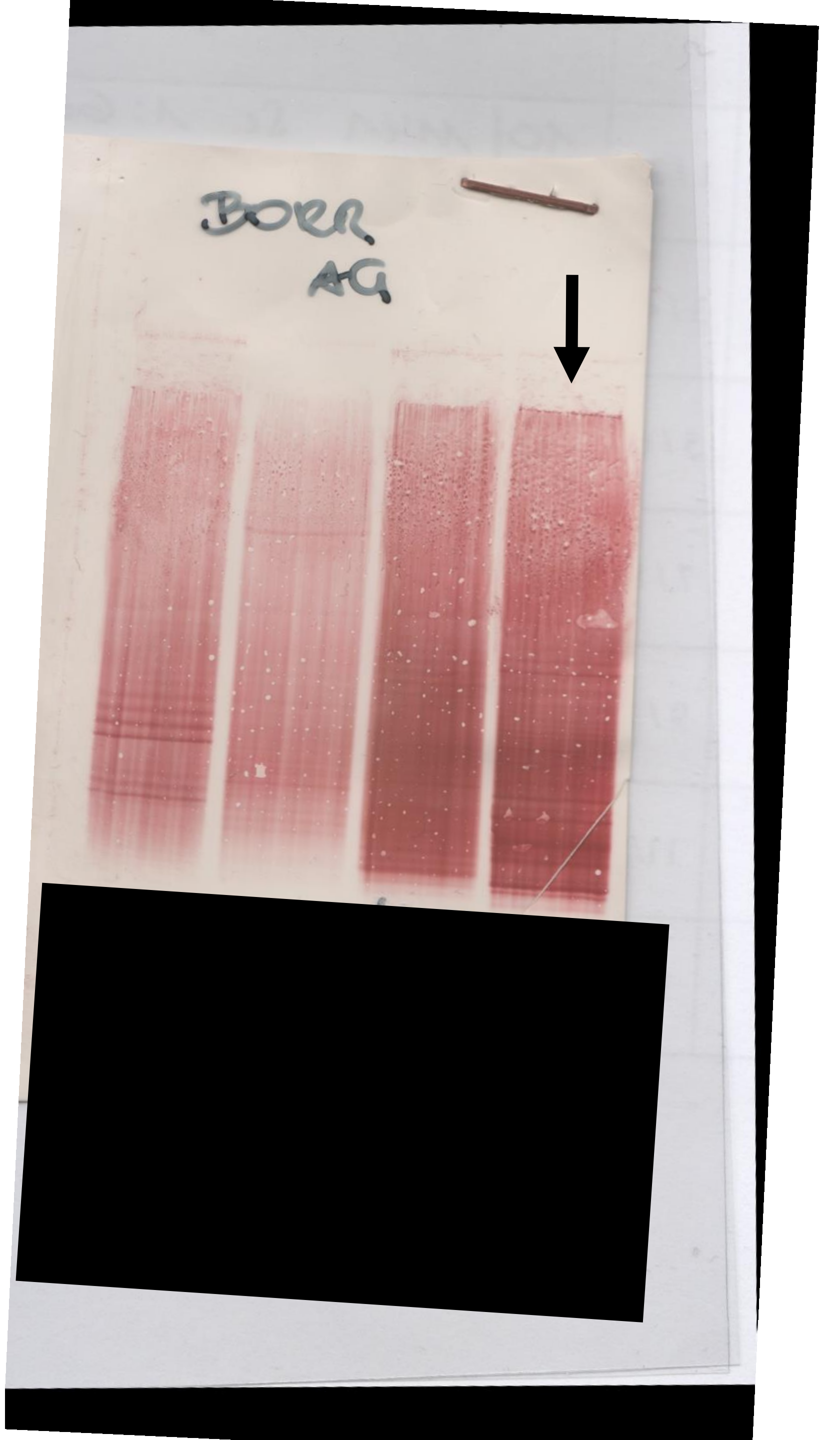

NB patient #8 uncoated

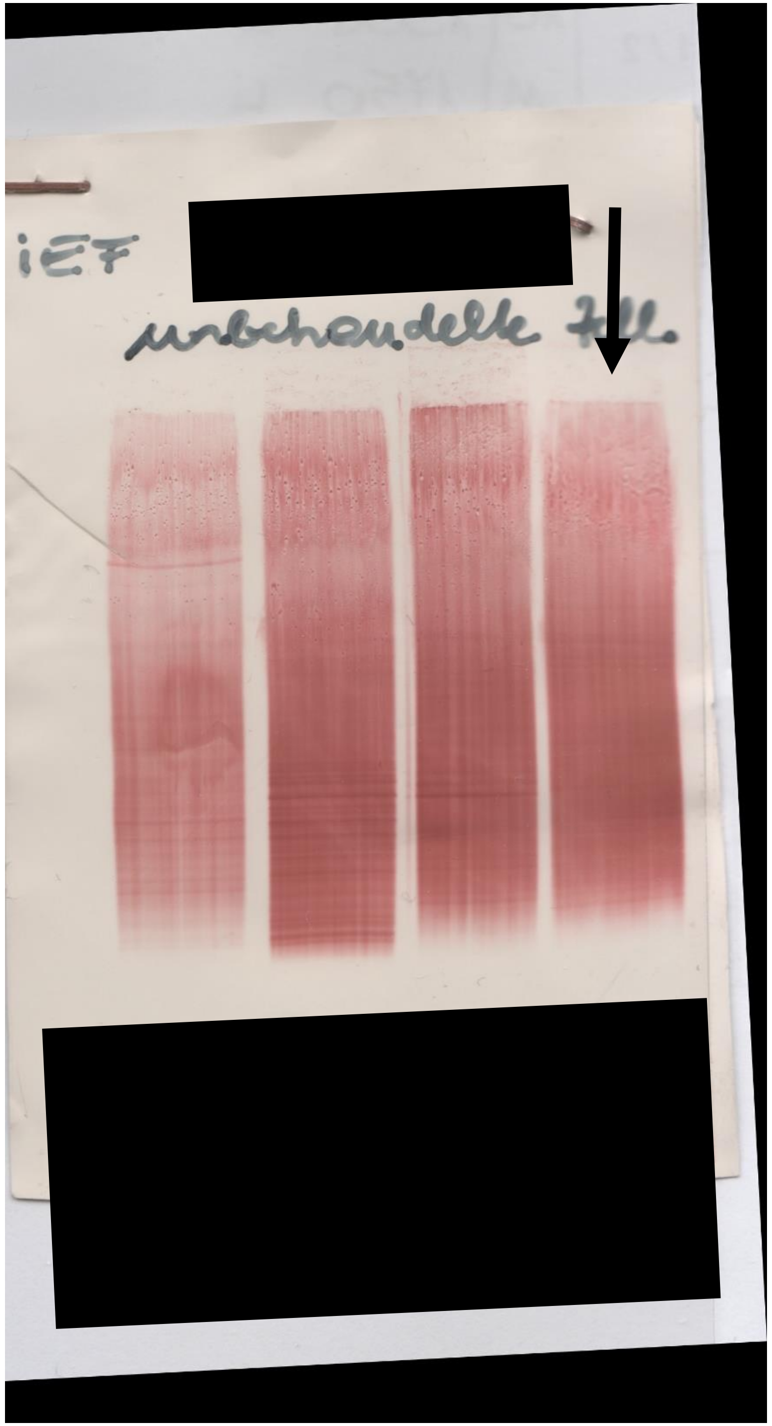

NB patient #8 precoated

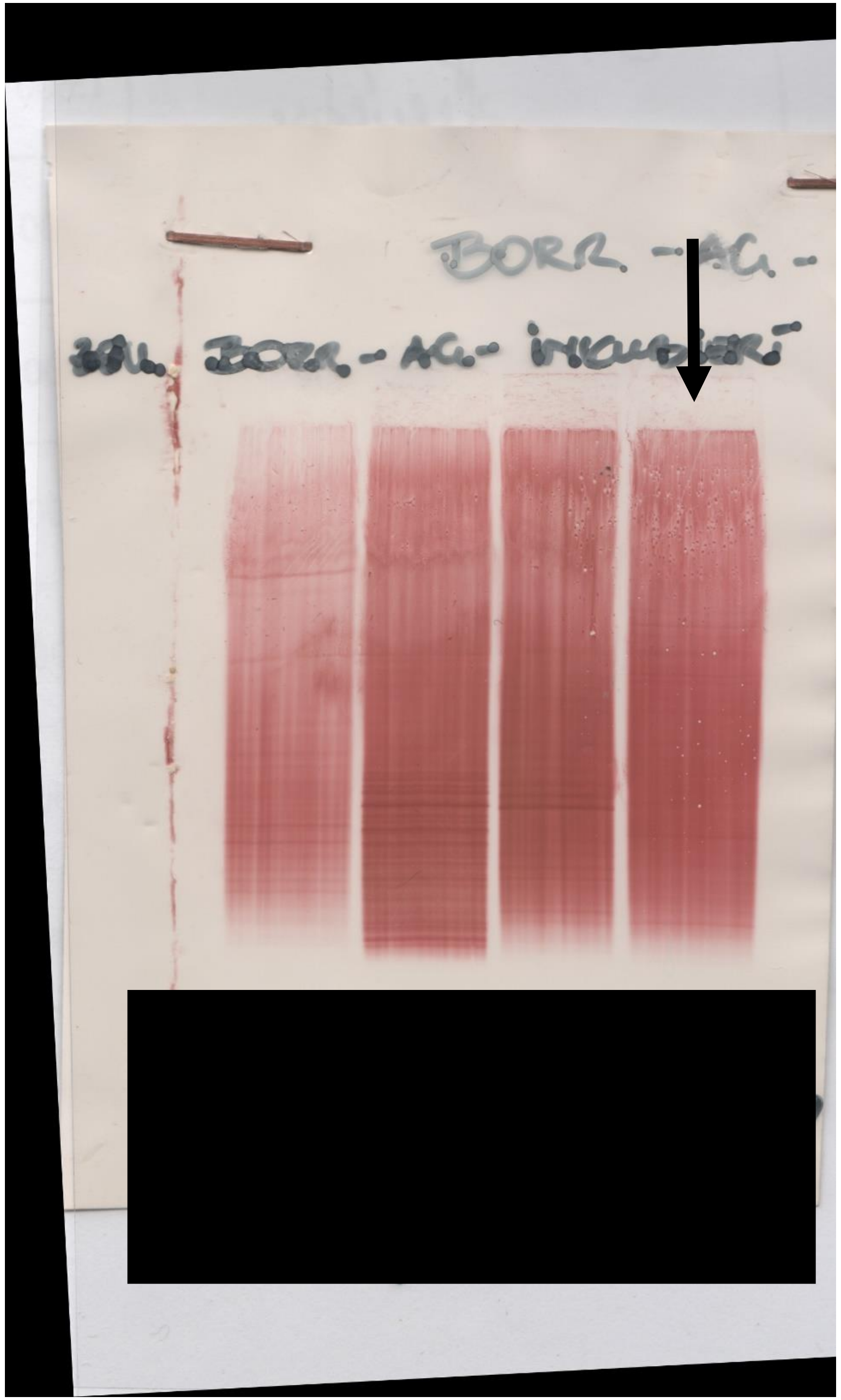

NB patient #9 uncoated

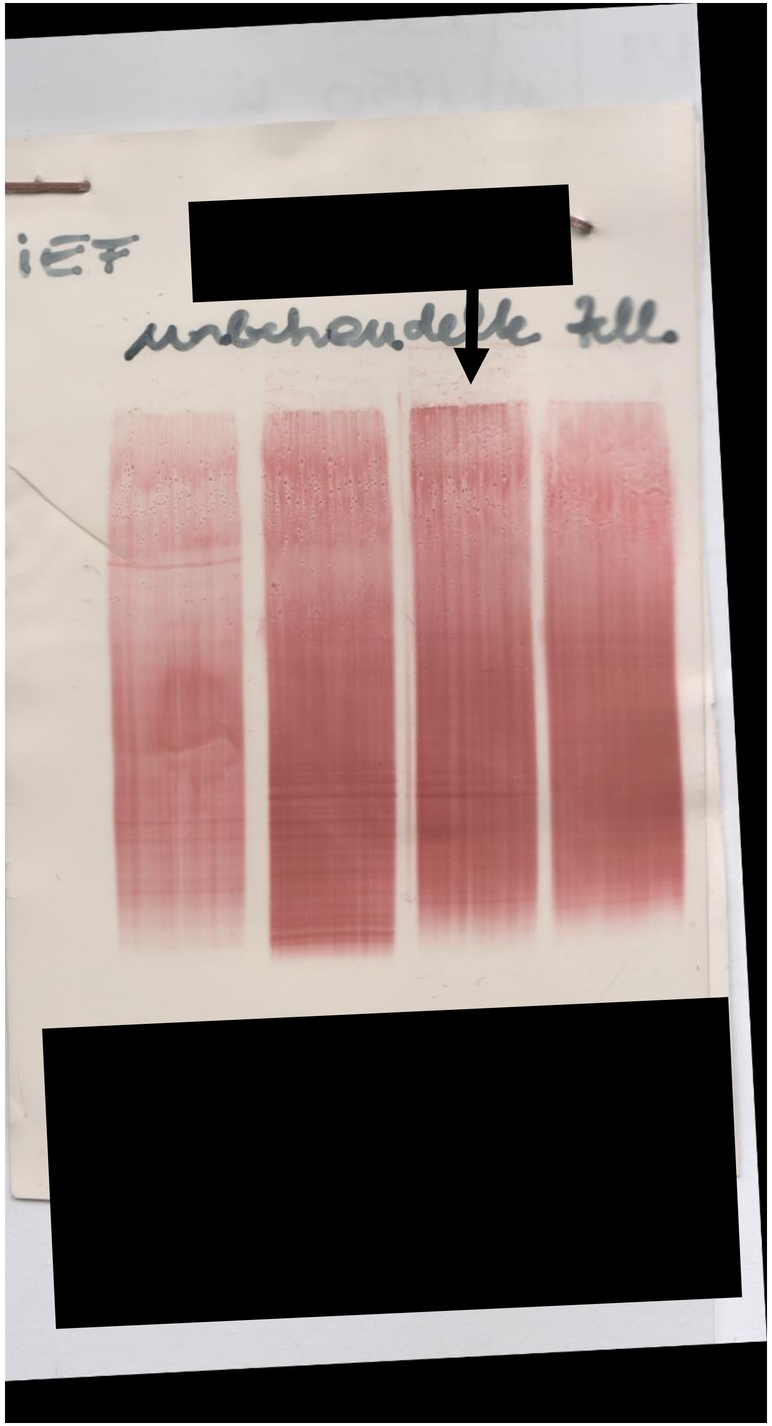

NB patient #9 precoated

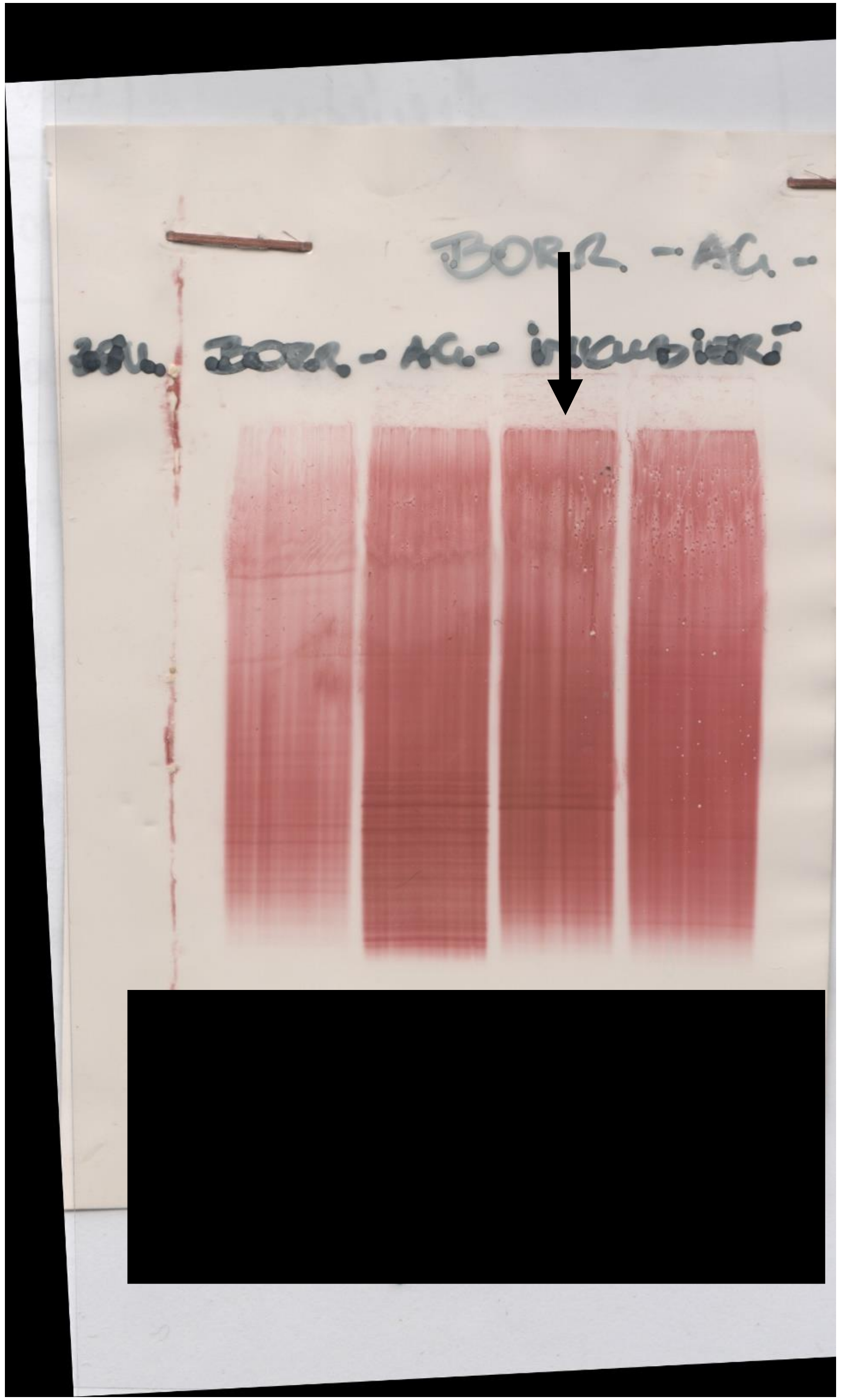

[redacted]

NB patient #10 uncoated

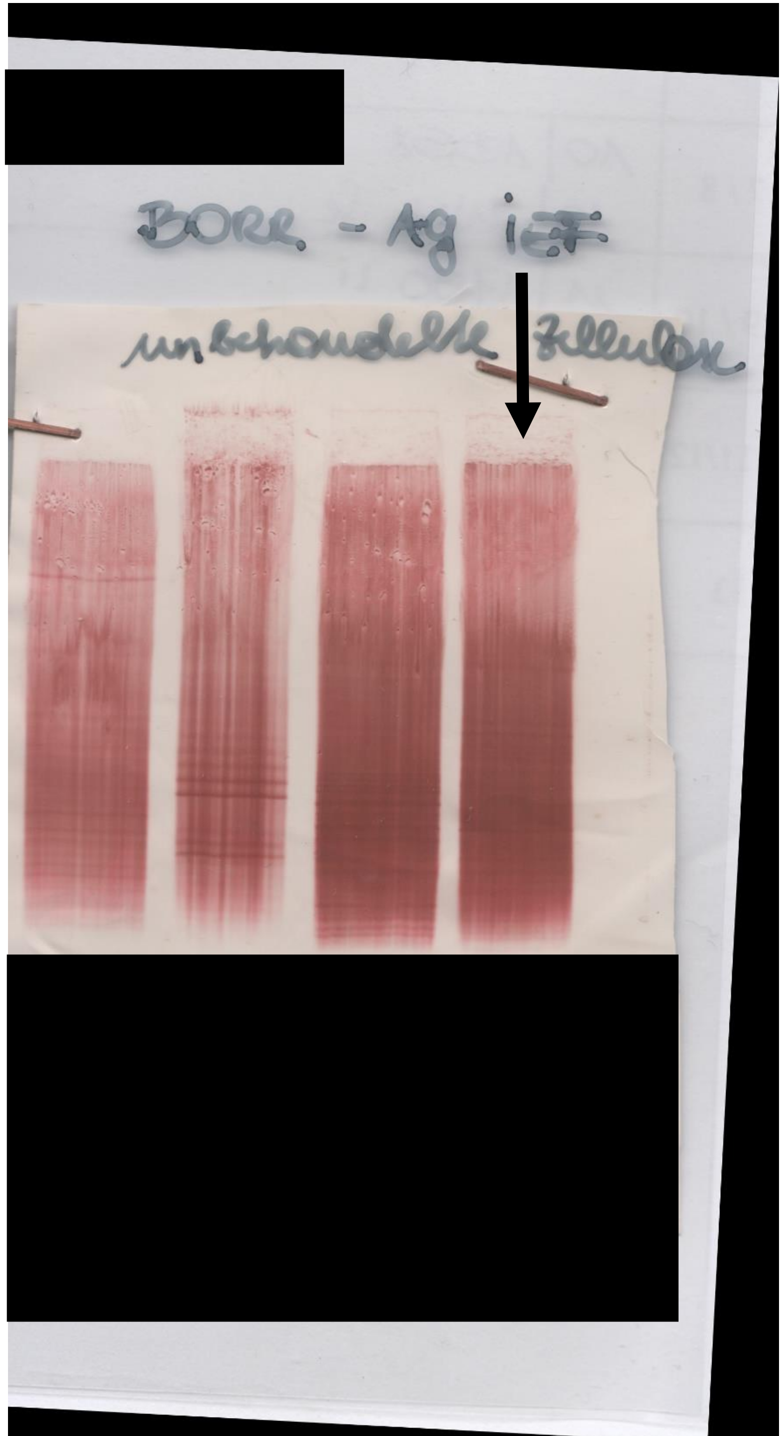

NB patient #10 precoated

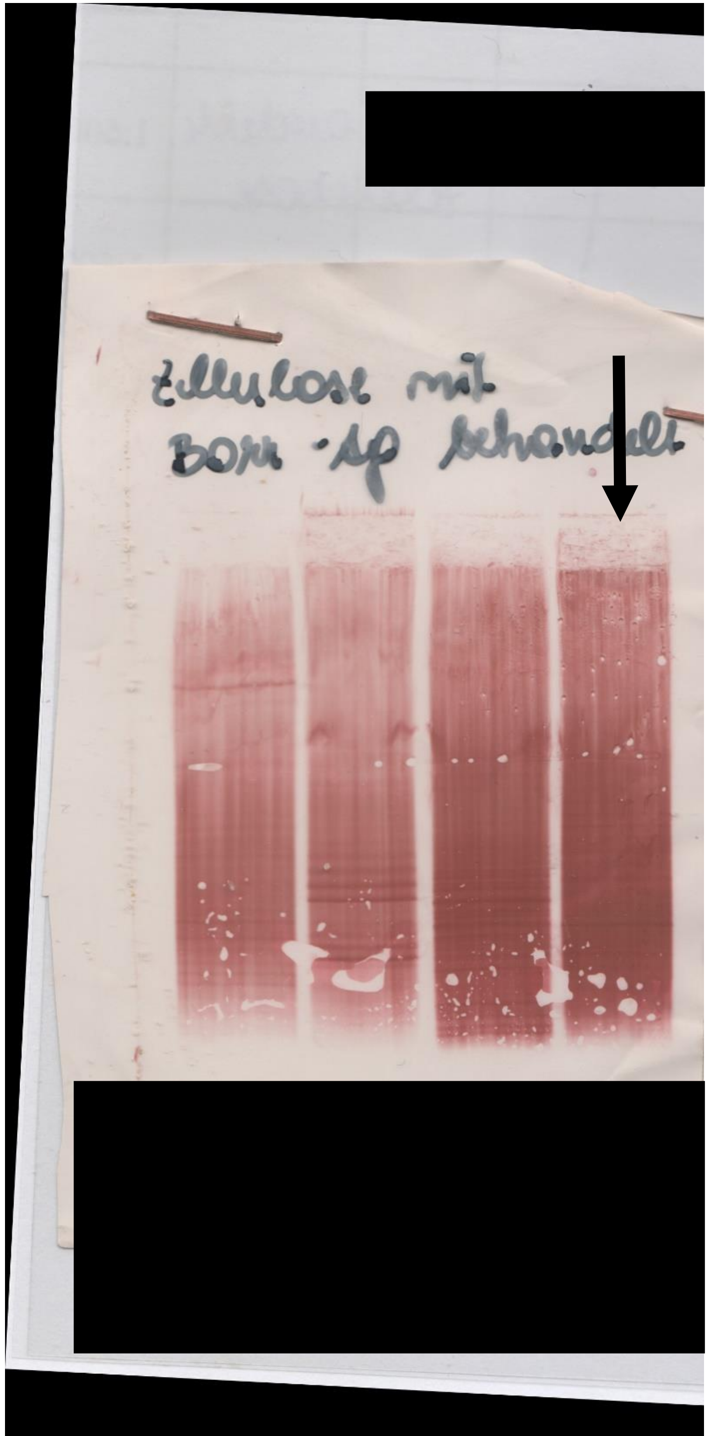

Control patient #1 uncoated

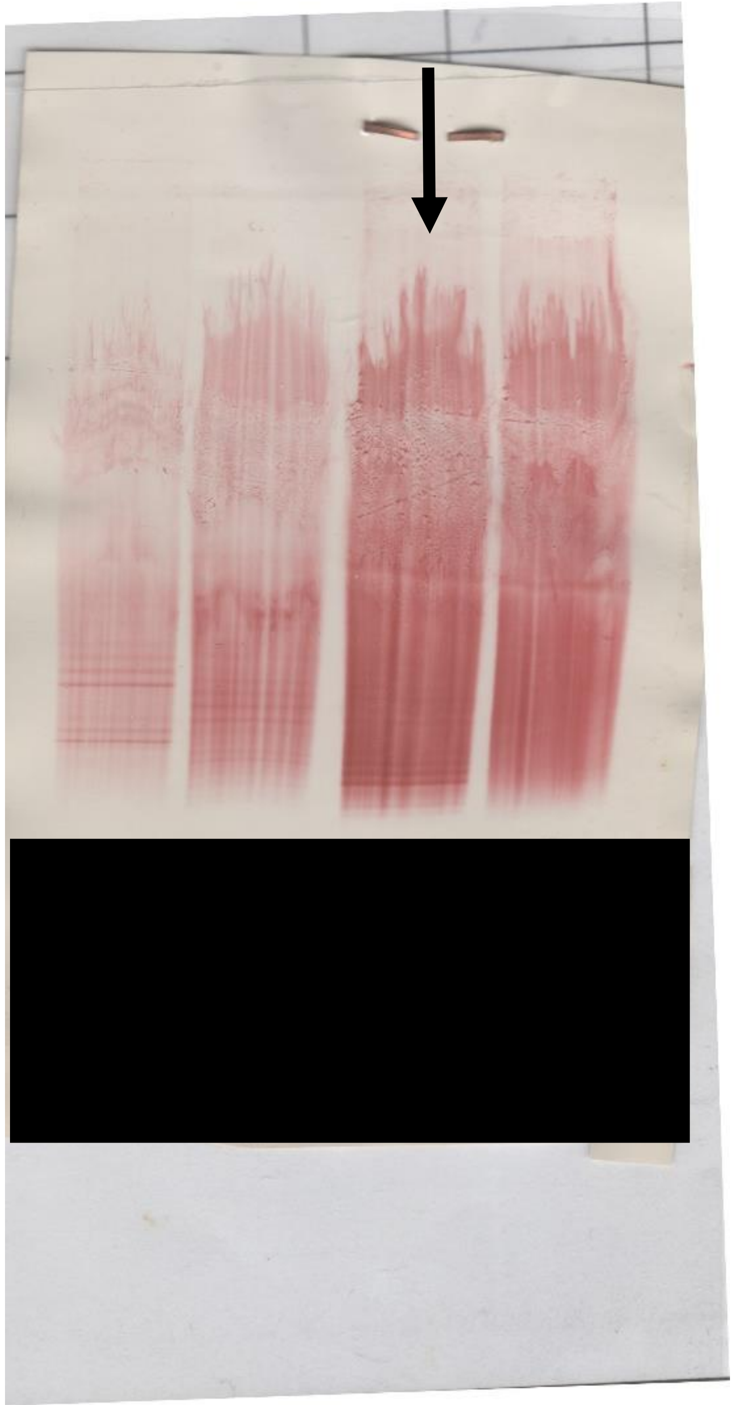

Control patient #1 precoated

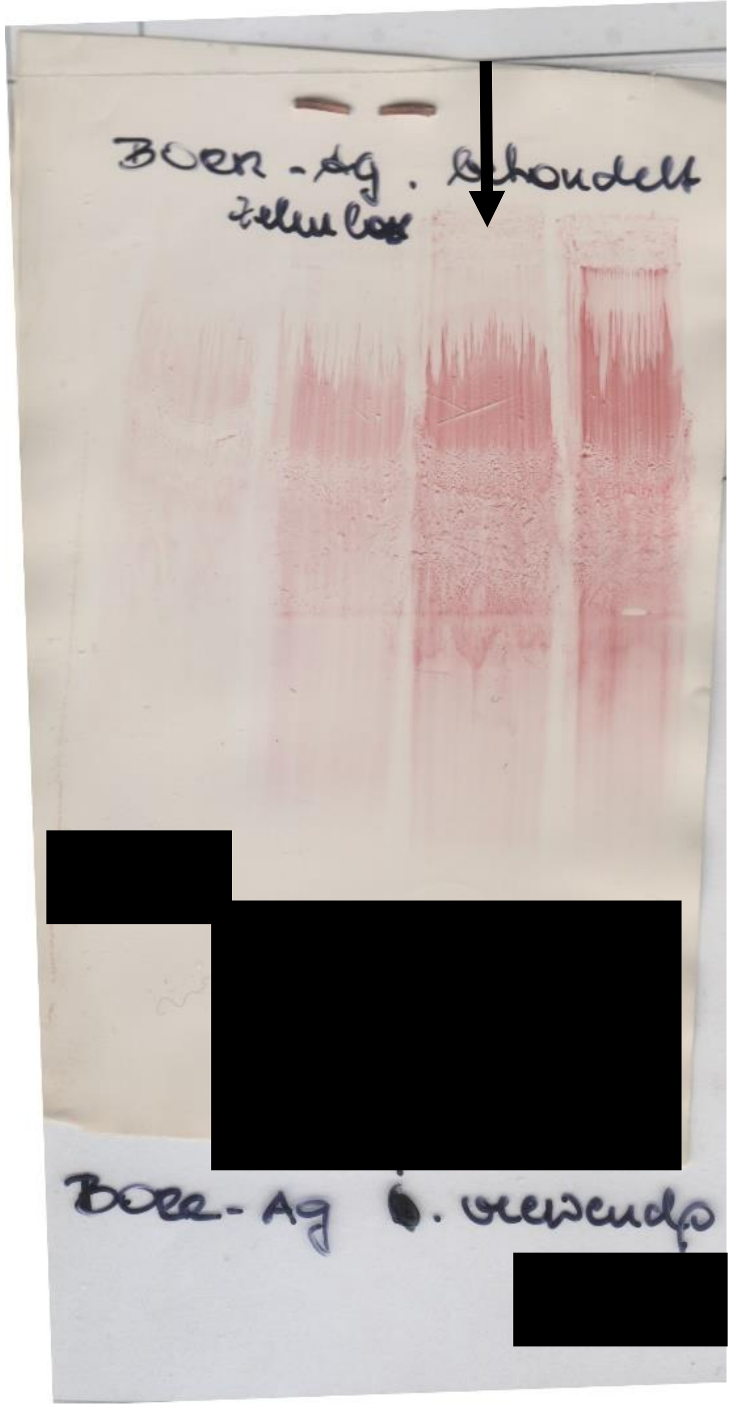

Control patient #2 uncoated

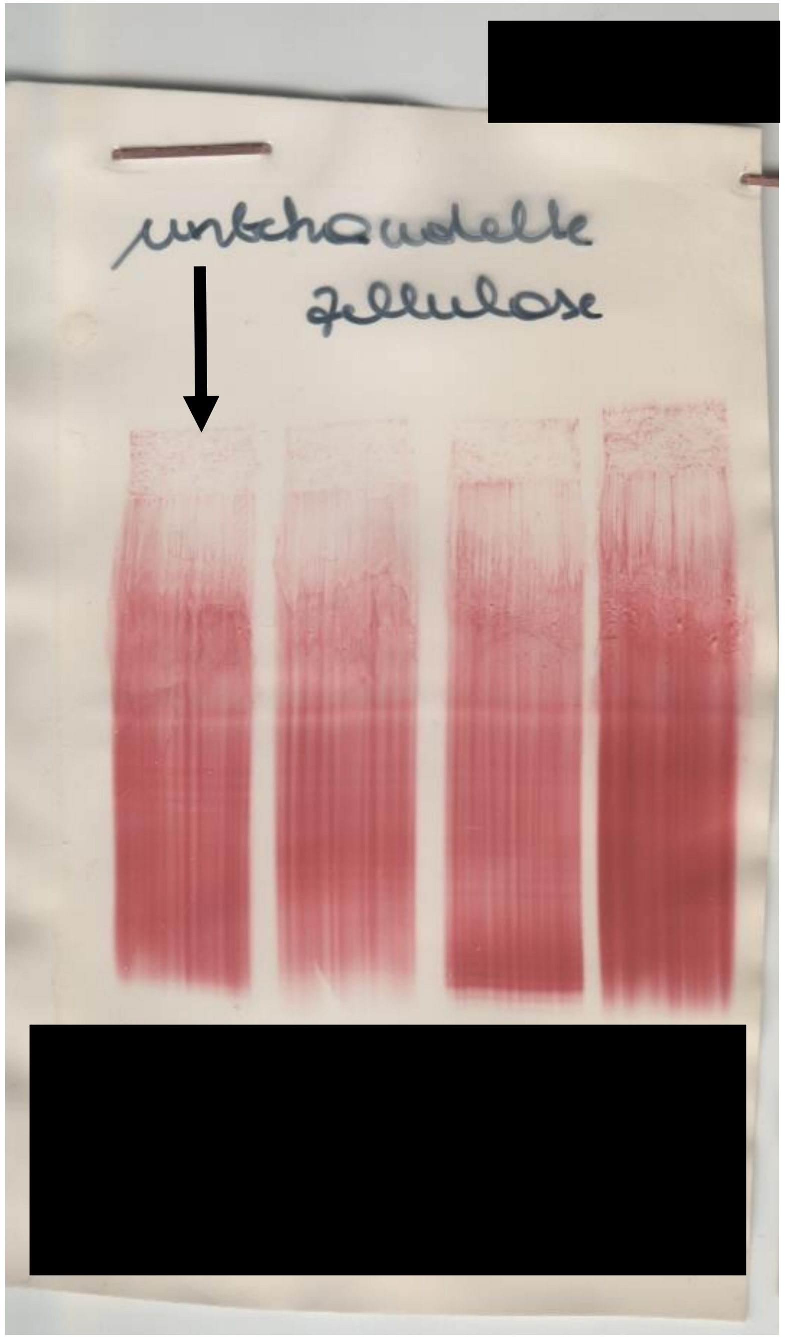

Control patient #2 precoated

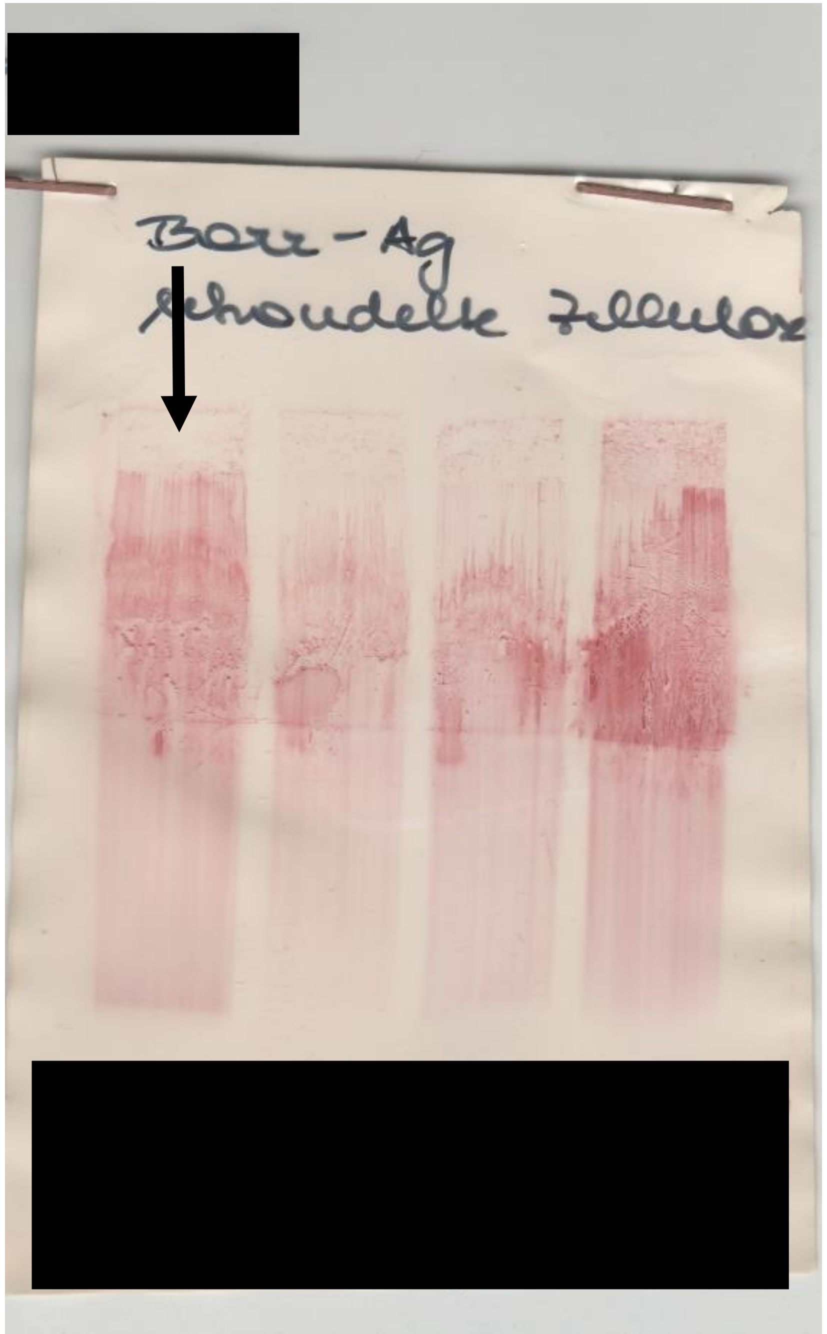

Control patient #3 uncoated

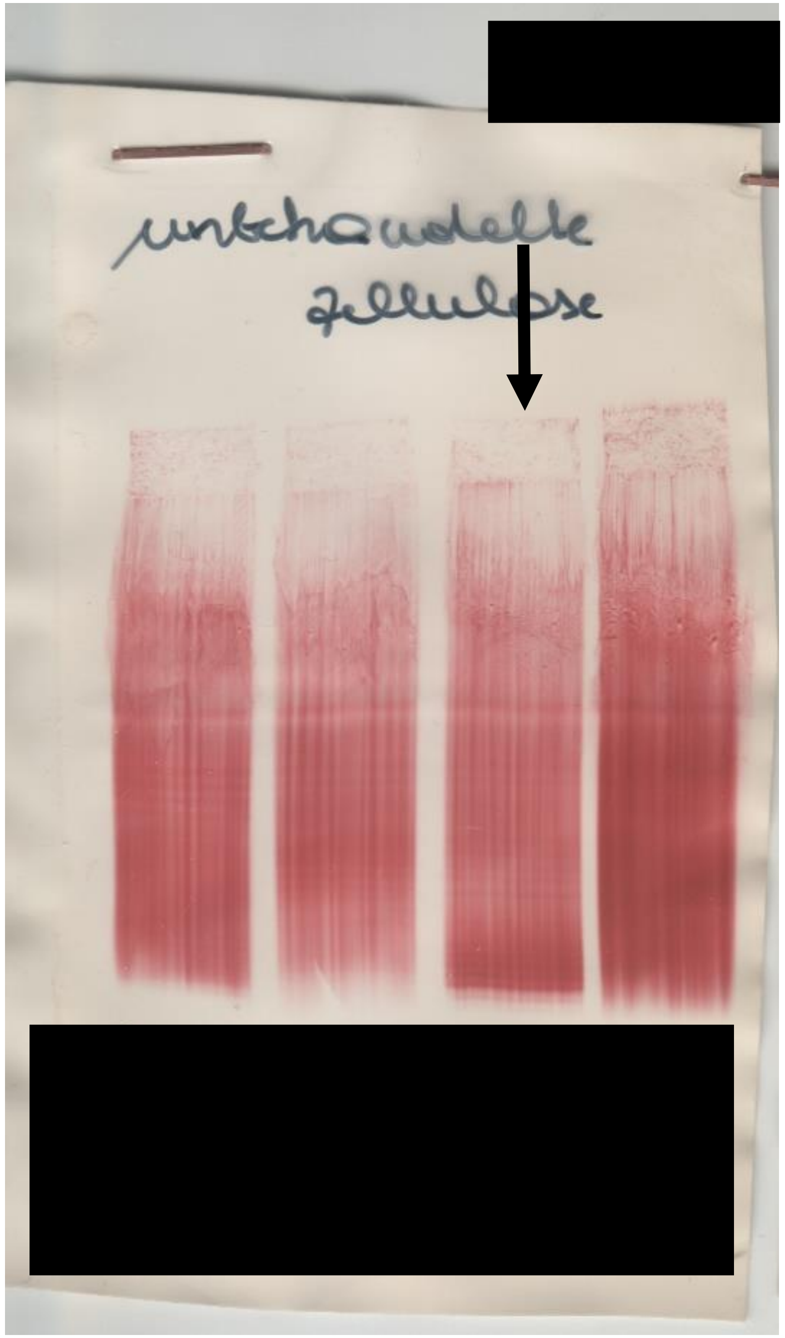

Control patient #3 precoated

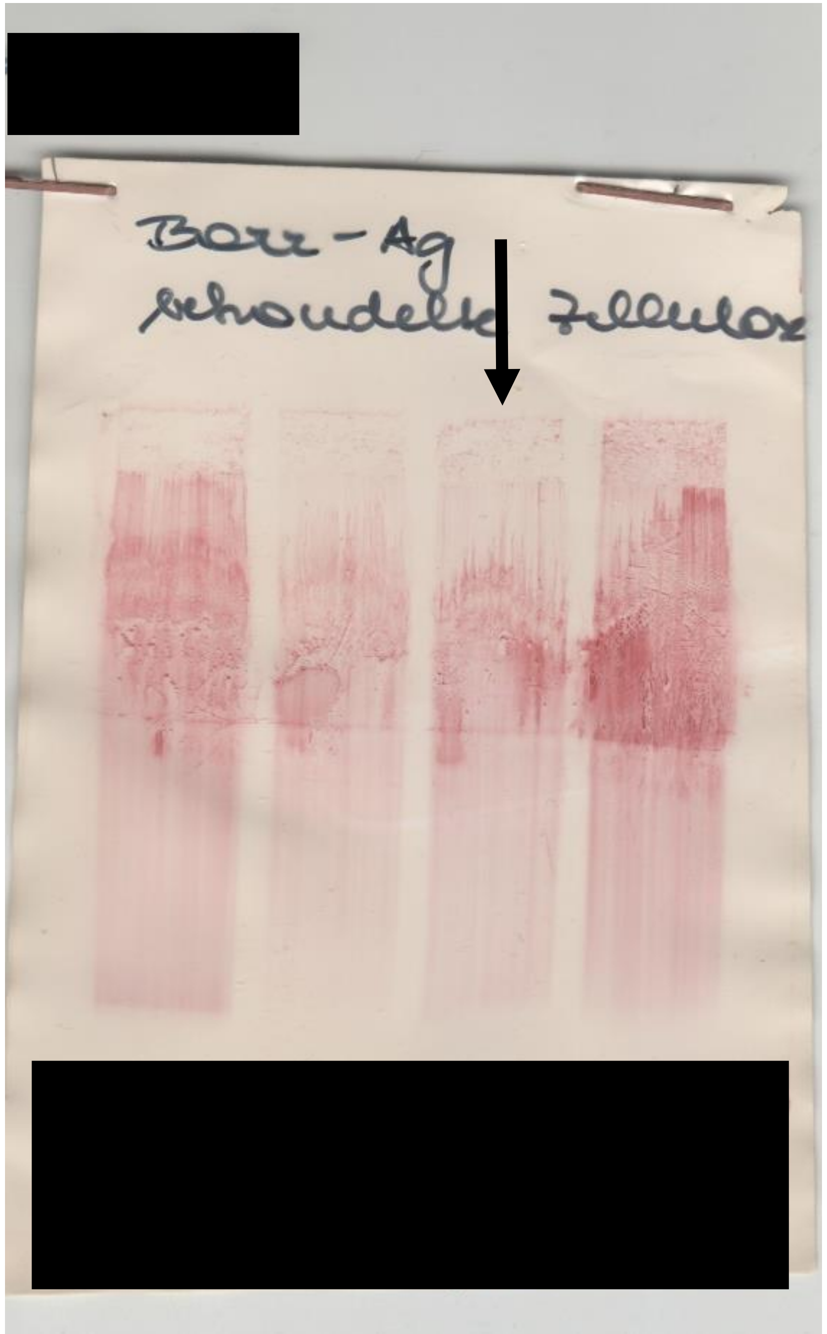

Control patient #4 uncoated

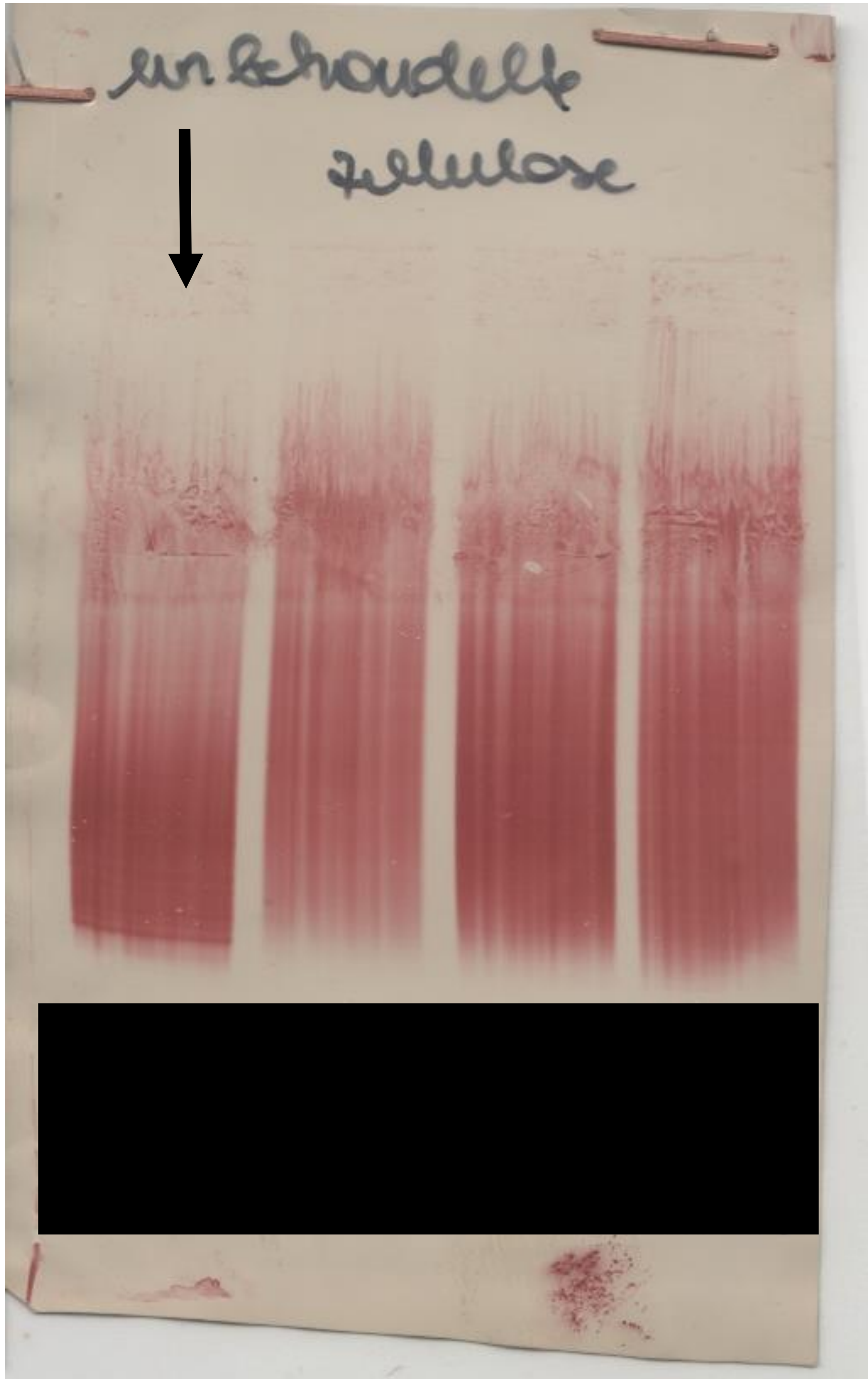

Control patient #4 precoated

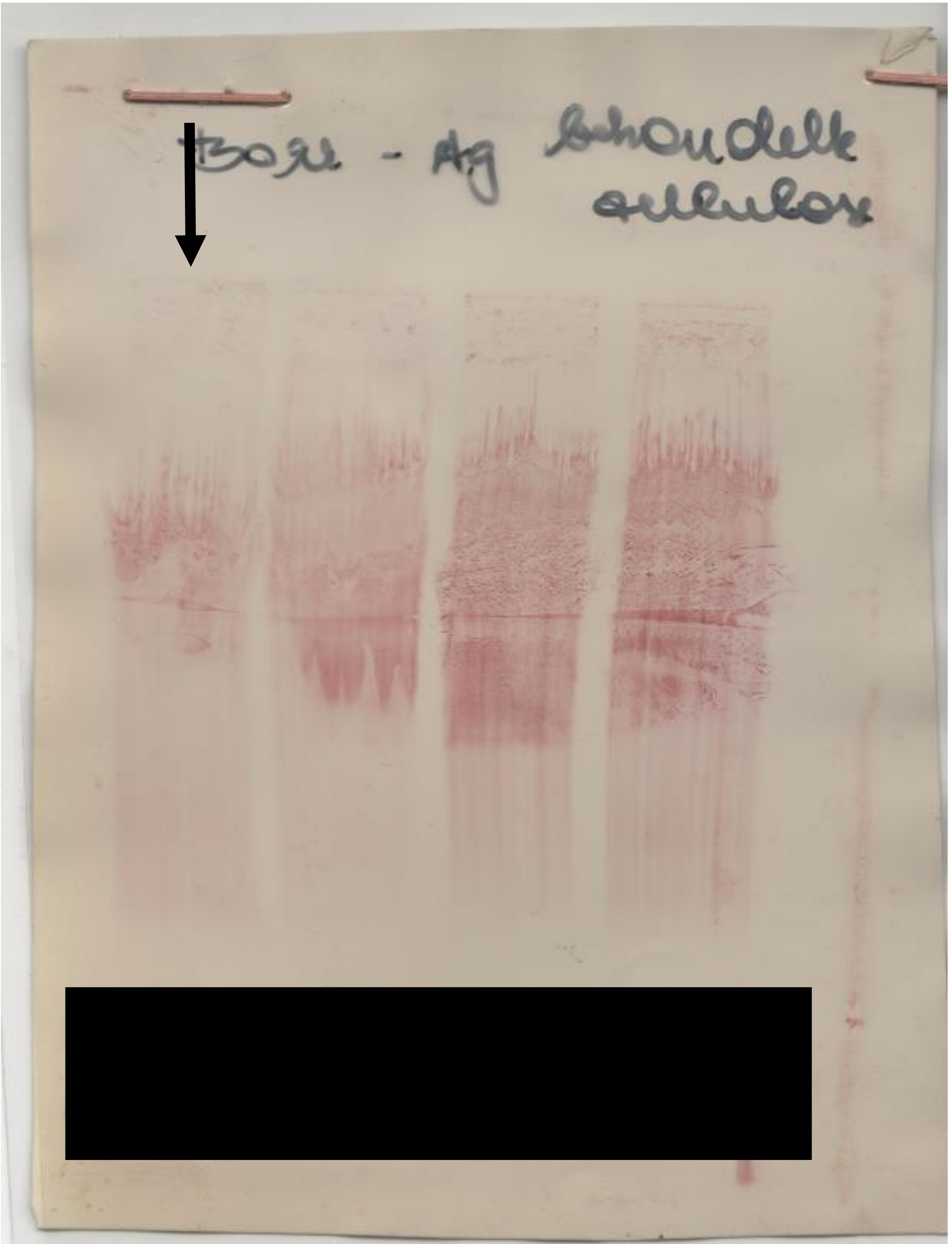

Control
patient #5
uncoated

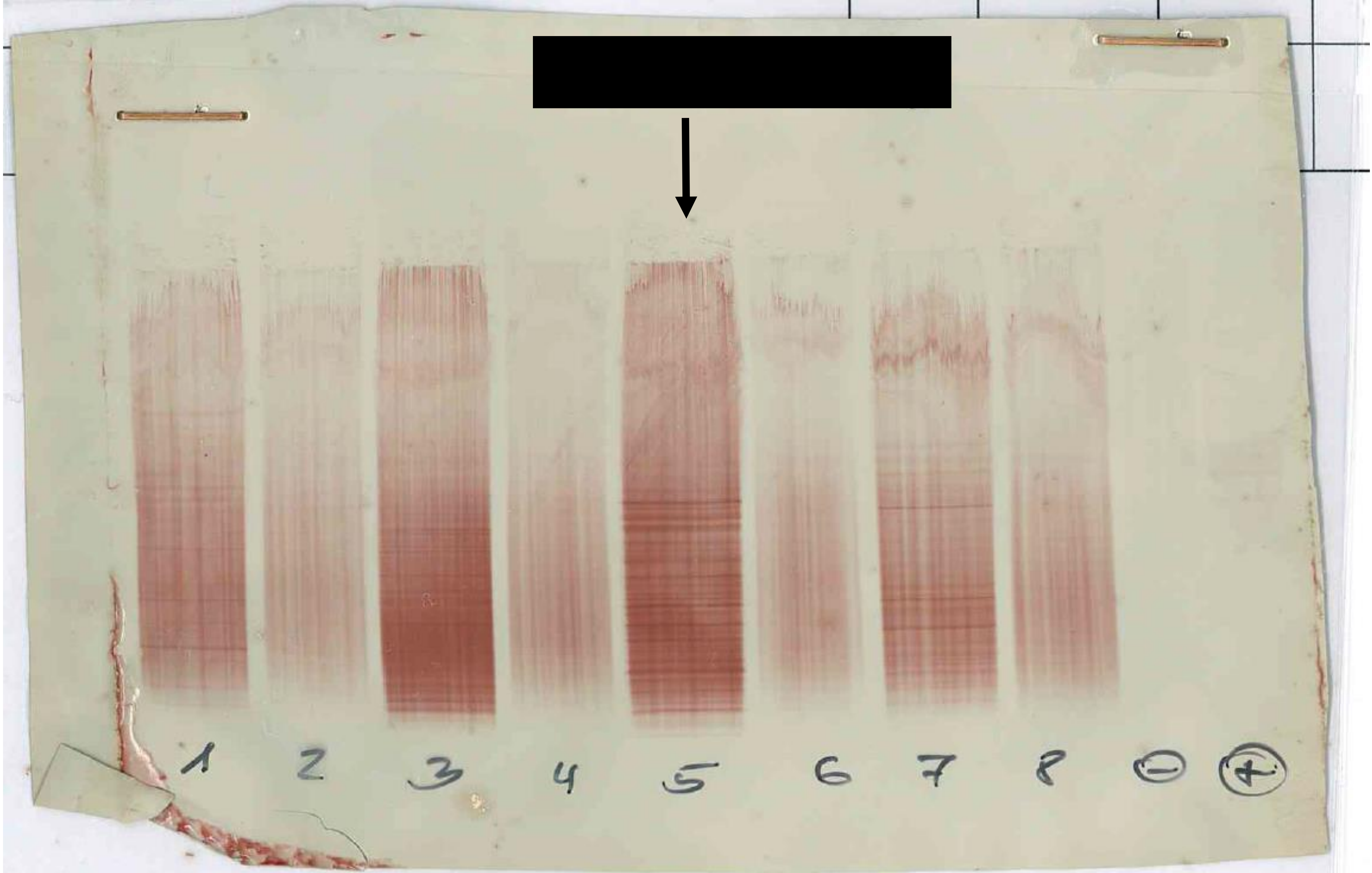

Control patient #6 uncoated

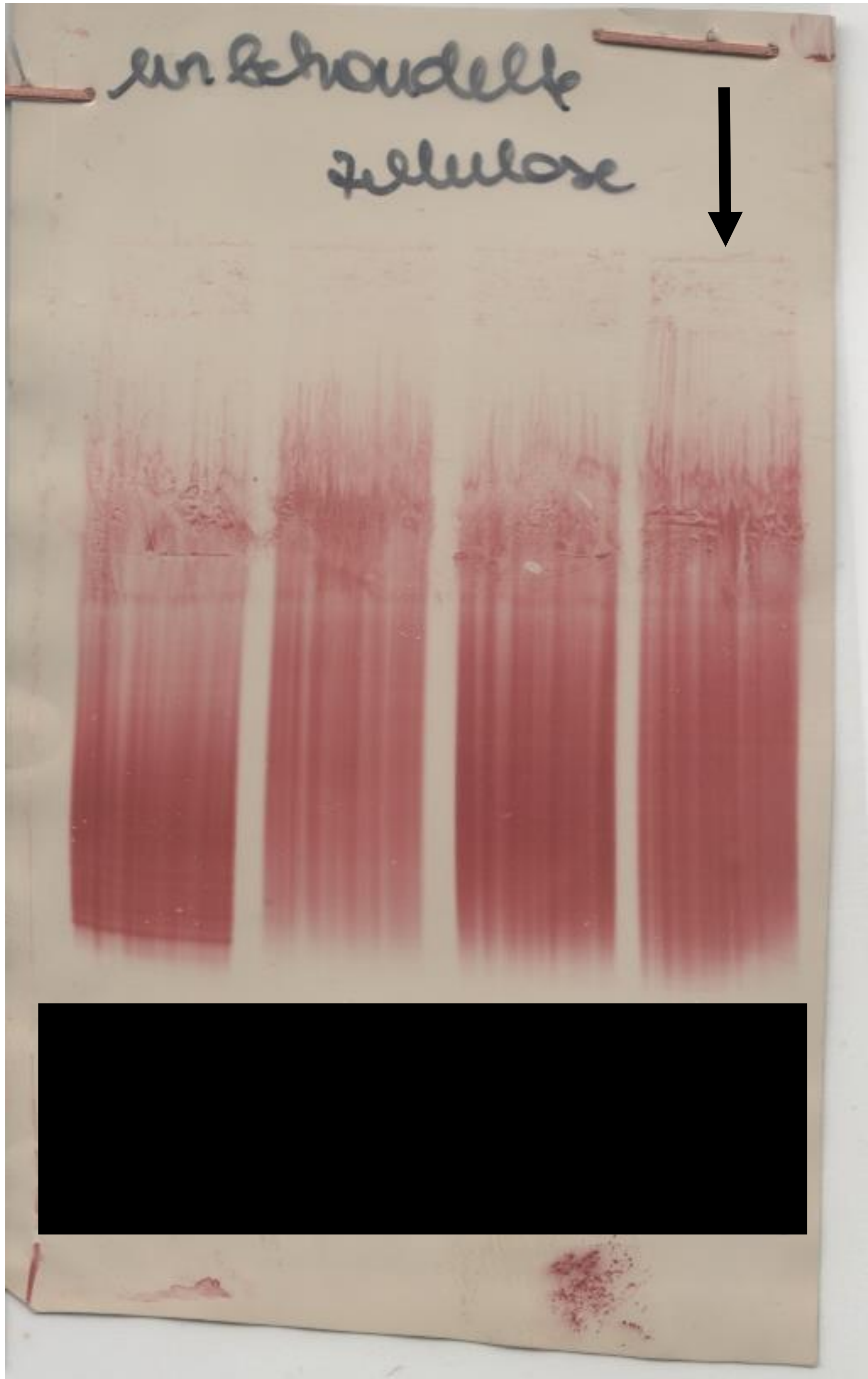

Control patient #6 precoated

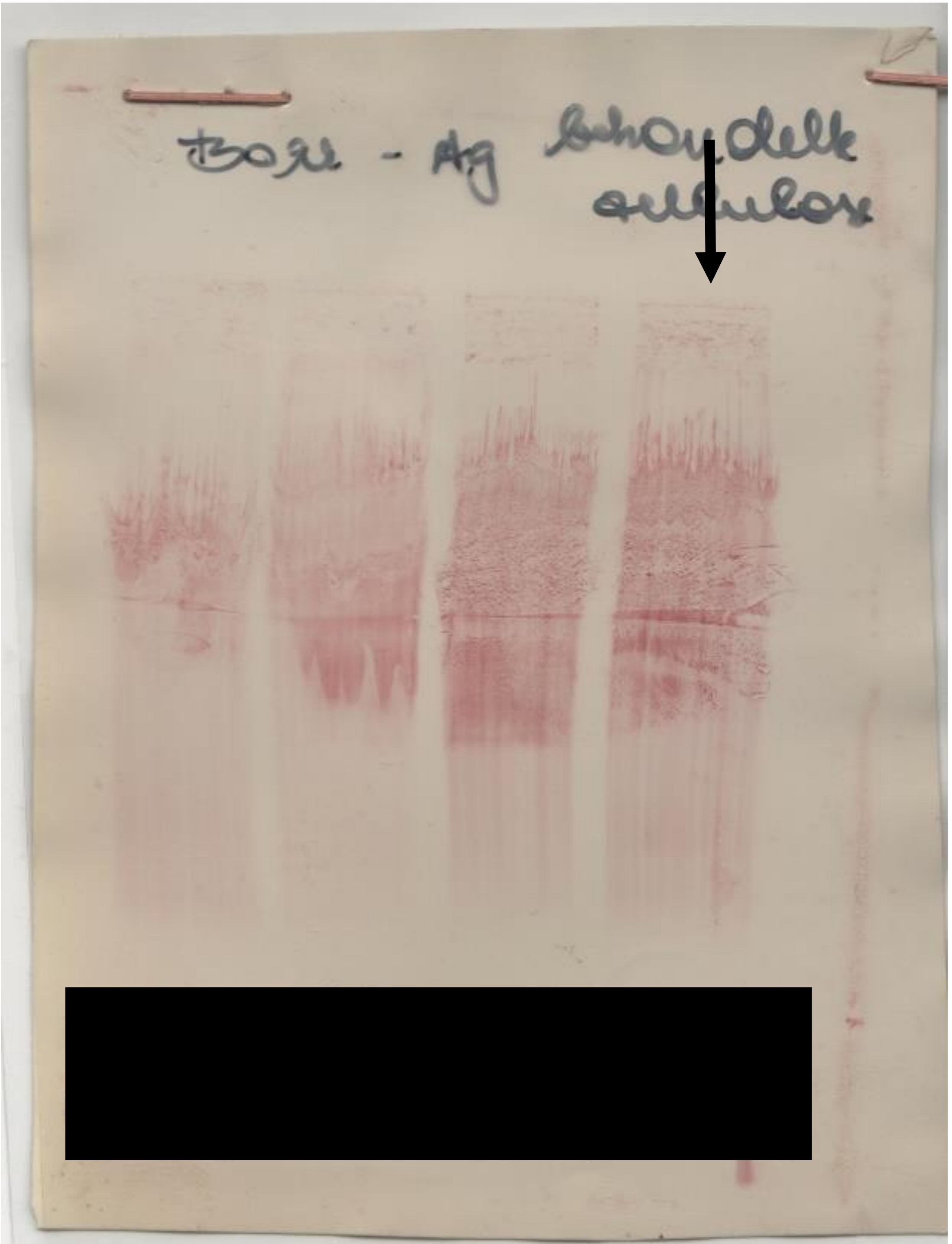

Control patient #7 uncoated

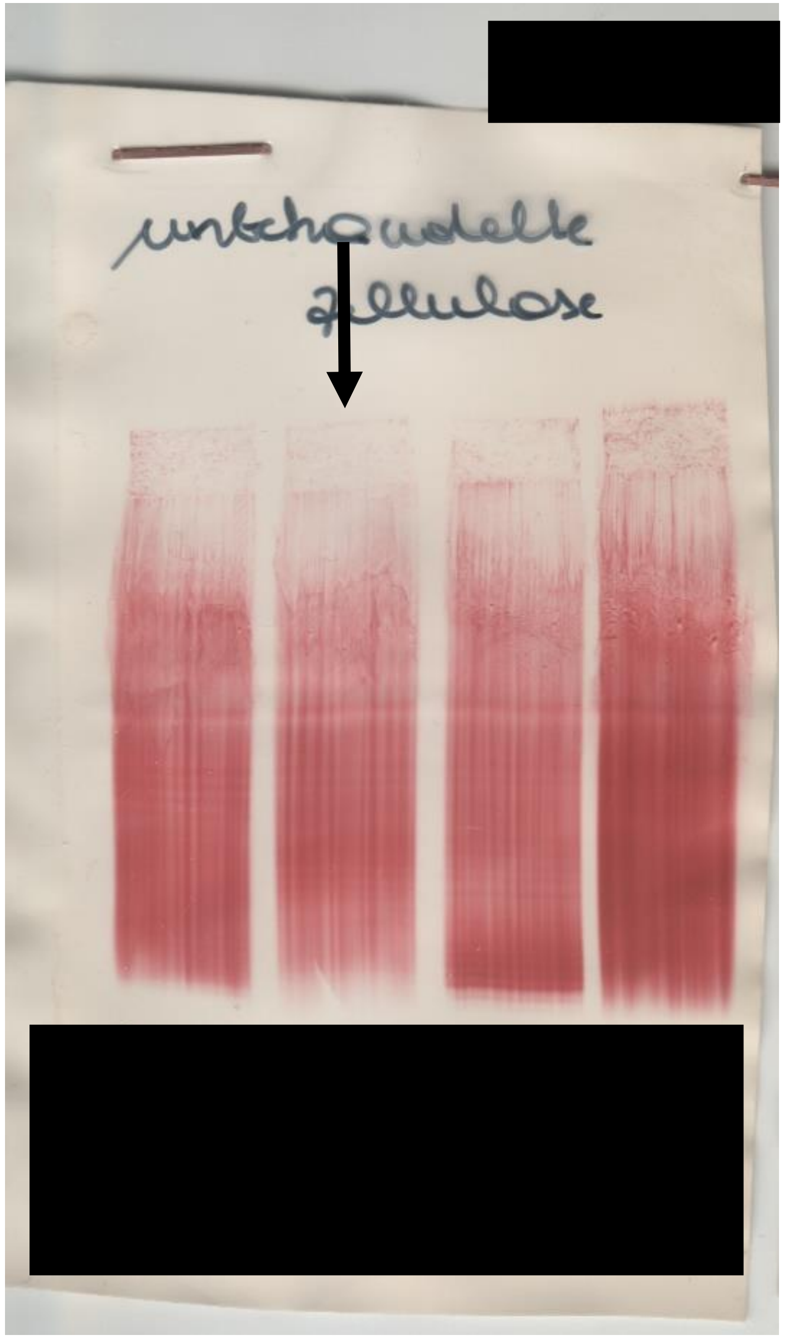

Control patient #7 precoated

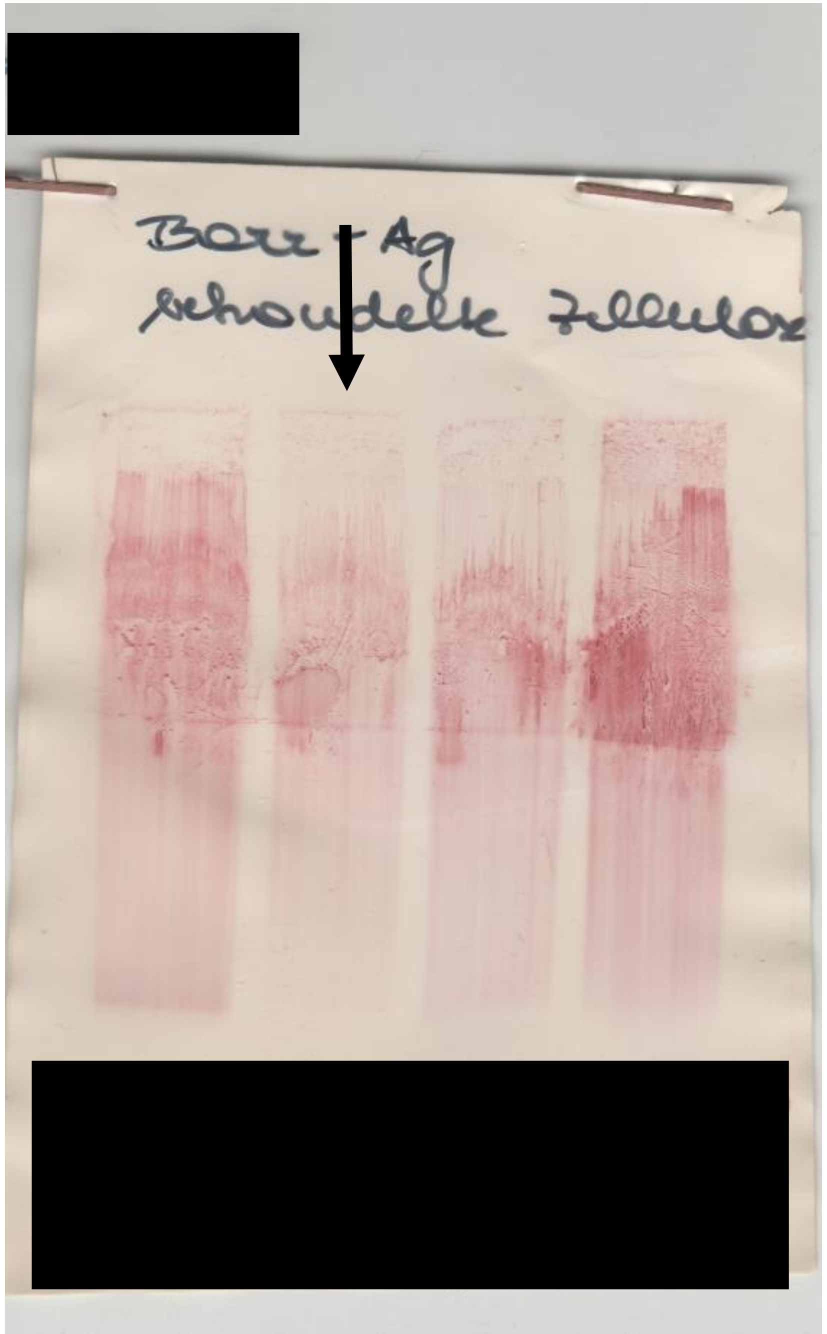

Control patient #8 uncoated

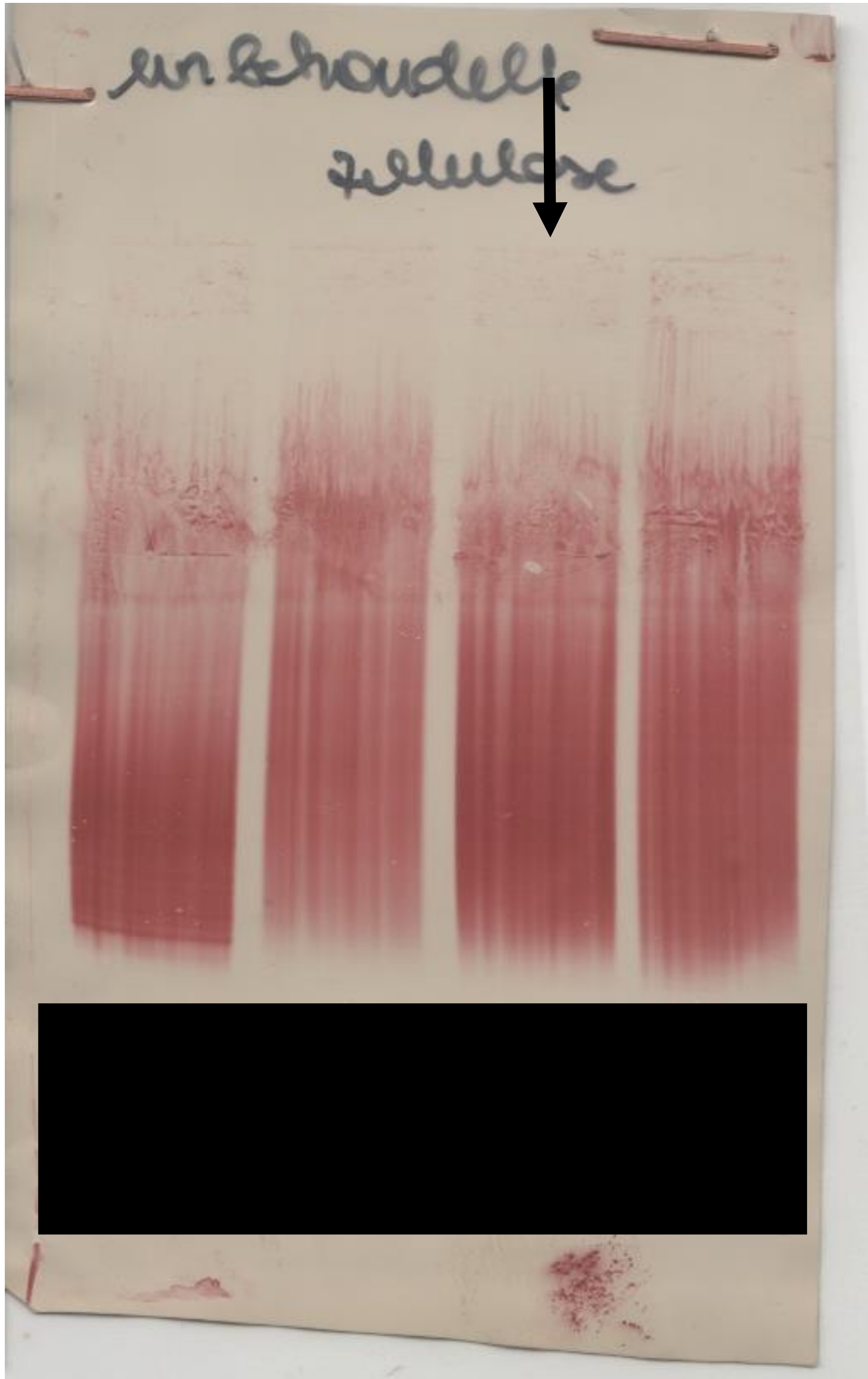

Control patient #8 precoated

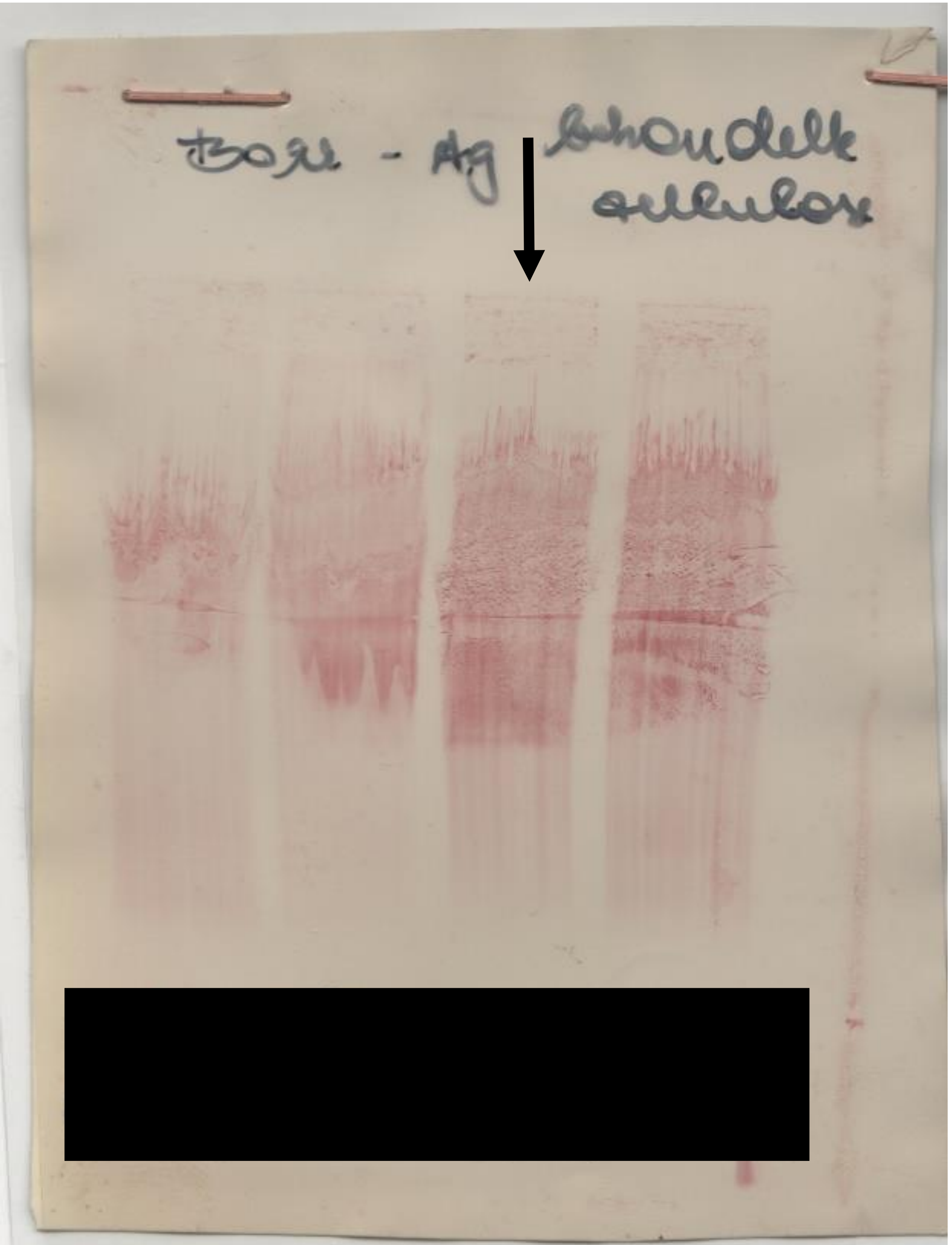

Control
patient #9
uncoated

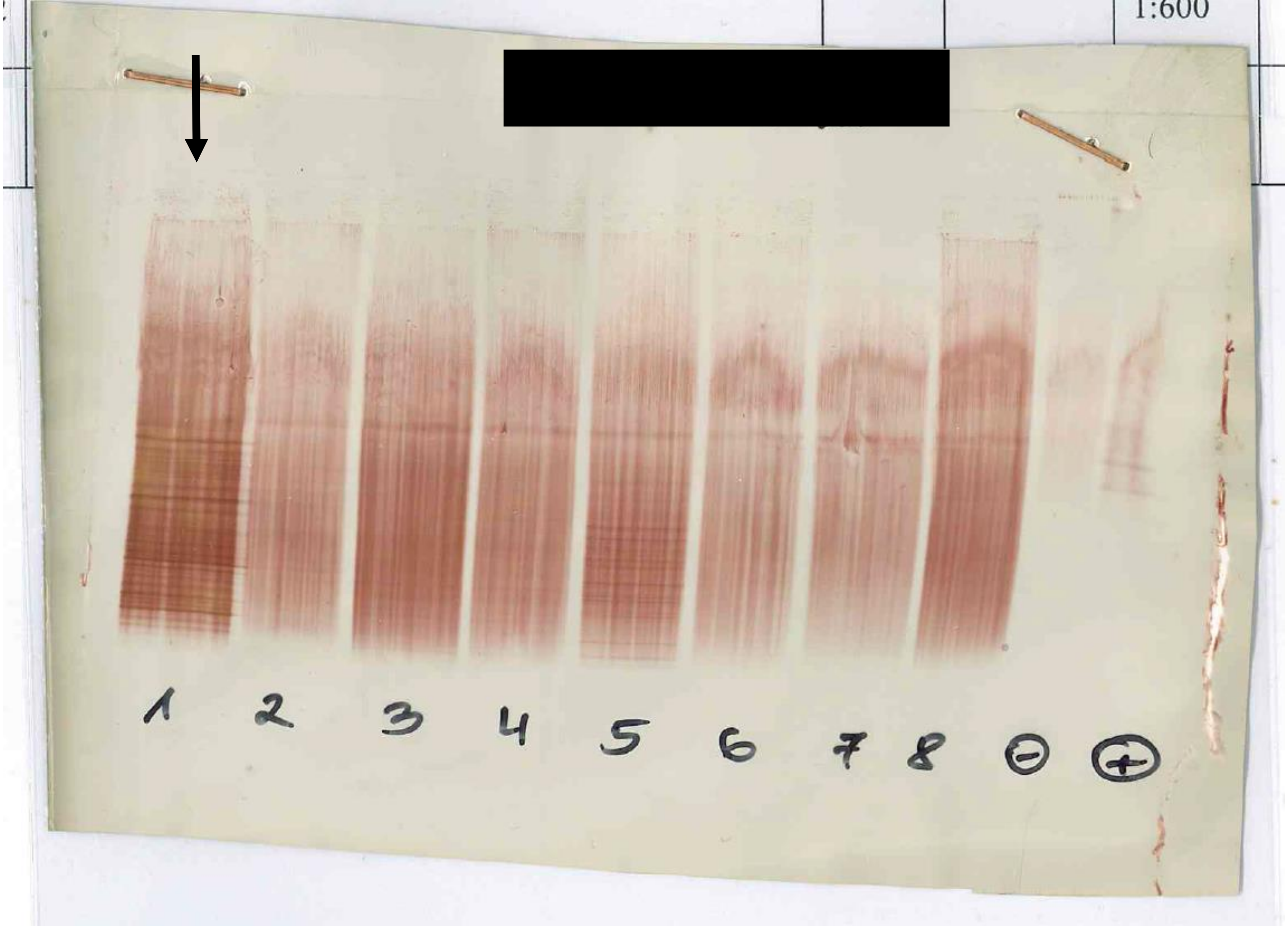

Control
patient
#10
uncoated

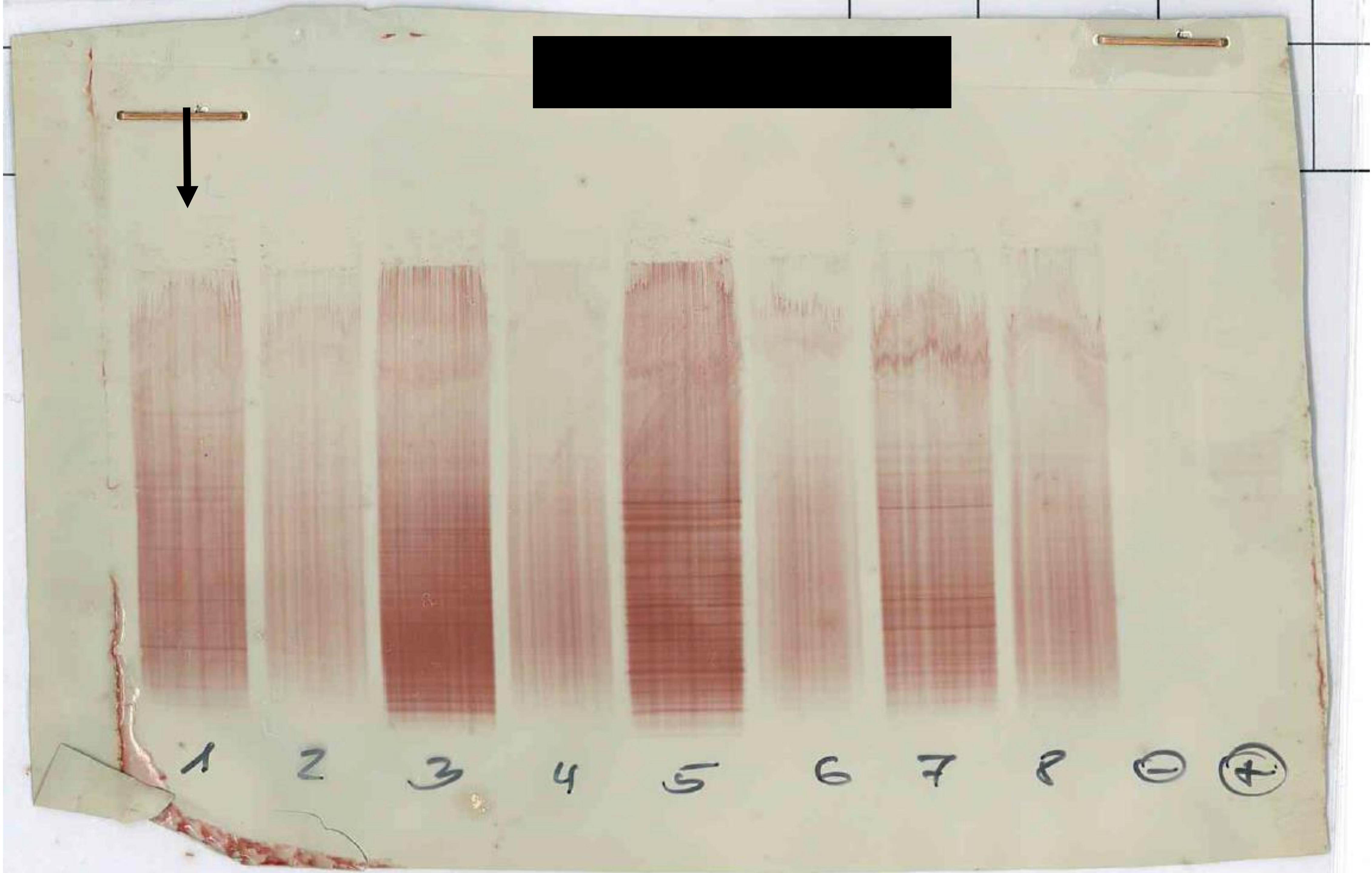

Control patient #11 uncoated

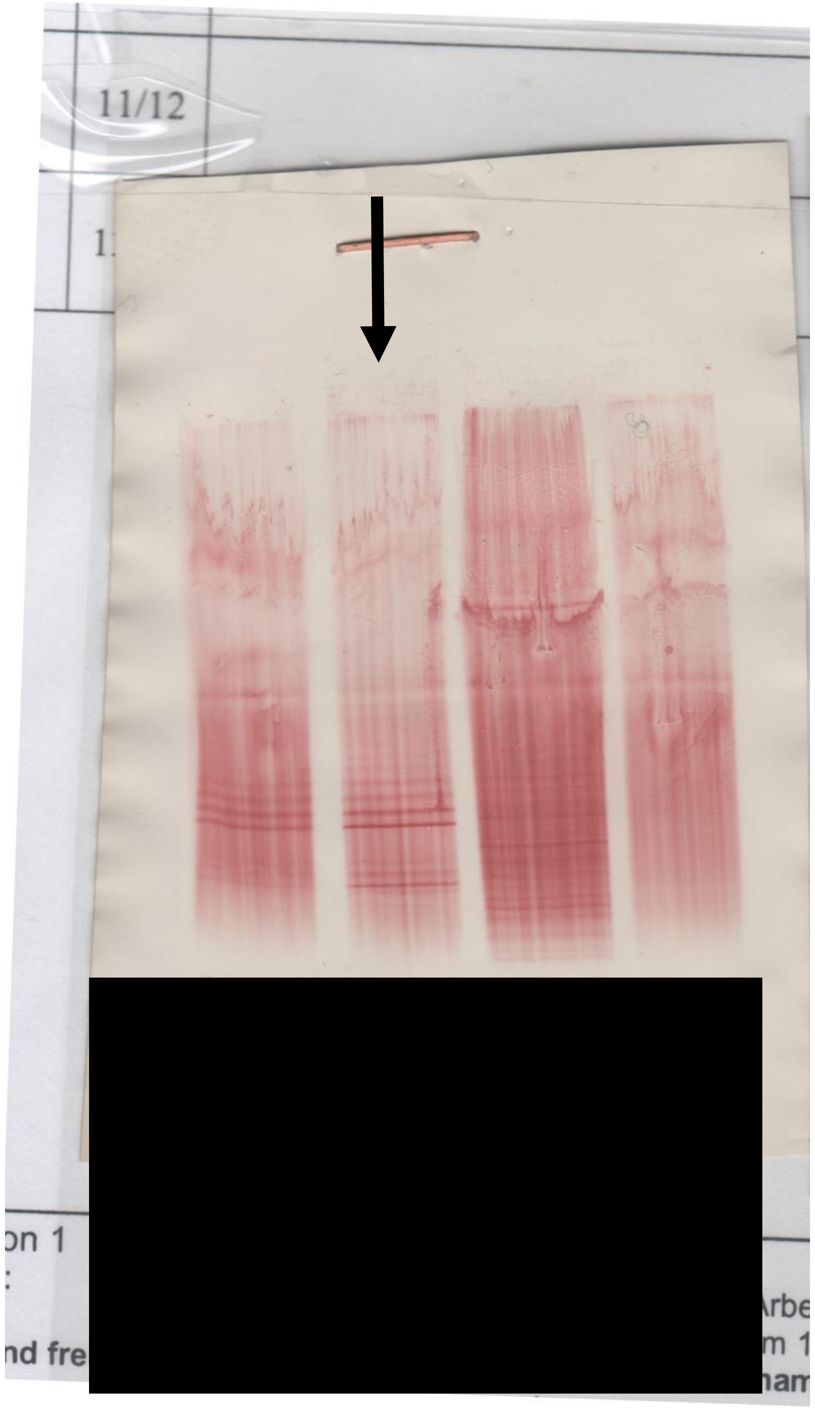

Control patient #11 precoated

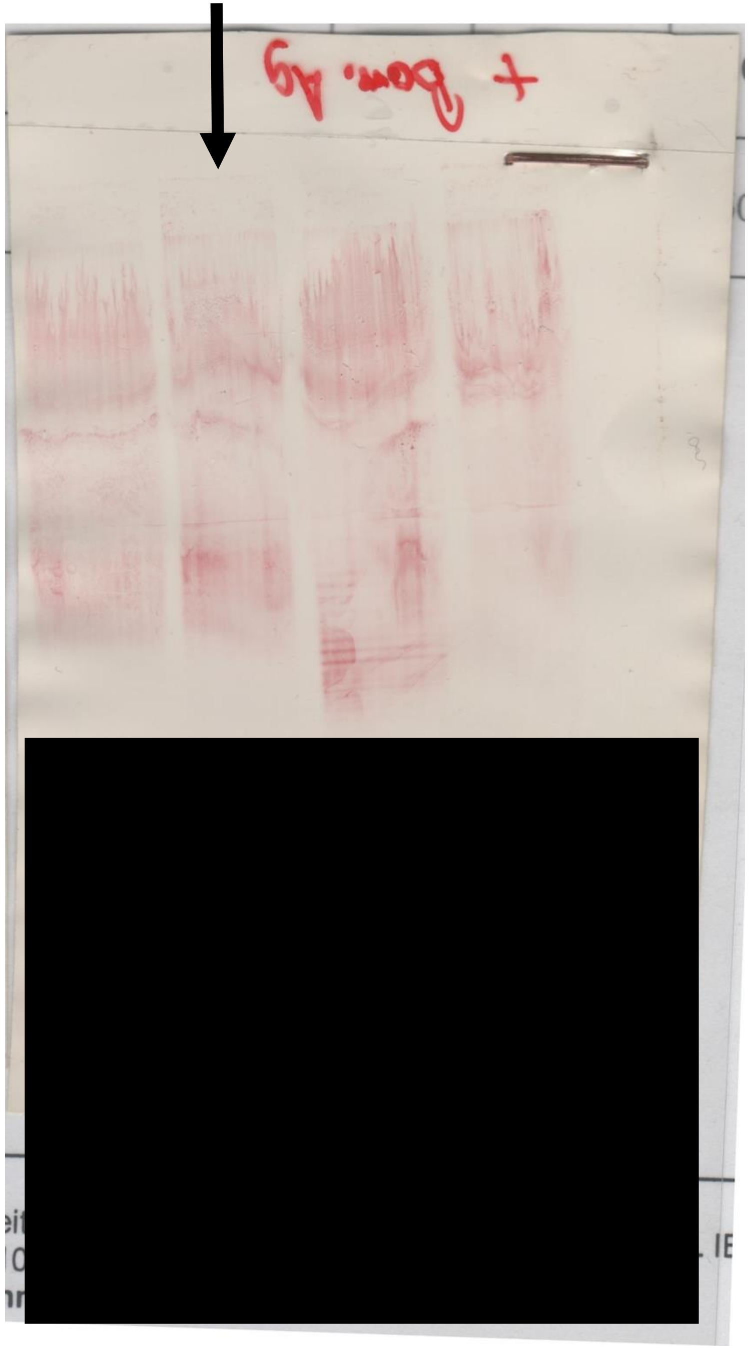

[redacted]
