# Supplementary material for: Cerebrospinal fluid oligoclonal bands in Neuroborreliosis are specific for Borrelia burgdorferi
Source: PLoS One. 2020 Sep 25;15(9):e0239453. doi: 10.1371/journal.pone.0239453 (PMC7518929; doi:10.1371/journal.pone.0239453)
Supplement: Attachment — Submitted filename: Response to Reviewers.docx [file pone.0239453.s006.docx]

**Journal Requirements**:

1. Please ensure that your manuscript meets PLOS ONE's style requirements, including those for file naming. The PLOS ONE style templates can be found at <https://journals.plos.org/plosone/s/file?id=wjVg/PLOSOne_formatting_sample_main_body.pdf> and <https://journals.plos.org/plosone/s/file?id=ba62/PLOSOne_formatting_sample_title_authors_affiliations.pdf>

Reply:

Uploaded files have been named as requested.

2.  PLOS ONE now requires that authors provide the original uncropped and unadjusted images underlying all blot or gel results reported in a submission’s figures or Supporting Information files. This policy and the journal’s other requirements for blot/gel reporting and figure preparation are described in detail at <https://journals.plos.org/plosone/s/figures#loc-blot-and-gel-reporting-requirements> and <https://journals.plos.org/plosone/s/figures#loc-preparing-figures-from-image-files>. When you submit your revised manuscript, please ensure that your figures adhere fully to these guidelines and provide the original underlying images for all blot or gel data reported in your submission. See the following link for instructions on providing the original image data: <https://journals.plos.org/plosone/s/figures#loc-original-images-for-blots-and-gels>.

In your cover letter, please note whether your blot/gel image data are in Supporting Information or posted at a public data repository, provide the repository URL if relevant, and provide specific details as to which raw blot/gel images, if any, are not available. Email us at [plosone@plos.org](mailto:plosone@plos.org) if you have any questions.

Reply:

We have added a supporting information file with the images used for analyses. However, the images are somewhat cropped because it is required for the image processing software which wouldn’t work otherwise. Nevertheless, we can confirm that the scans contain the whole region of interest, i.e. the area (lanes) where sample material got blotted on membranes.

3. Thank you for stating the following in the Financial Disclosure section:

'Borrelia antigens were provided free of charge by Euroimmun.
URL: [https://www.euroimmun.com/startseite.html](https://www.euroimmun.com/startseite.html)
The funders had no role in study design, data collection and analysis, decision to
publish, or preparation of the manuscript.'

Reply:

We have added this statement to the Financial Disclosures section.

We note that you received funding from a commercial source: Euroimmun

Please provide an amended Competing Interests Statement that explicitly states this commercial funder, along with any other relevant declarations relating to employment, consultancy, patents, products in development, marketed products, etc.

Within this Competing Interests Statement, please confirm that this does not alter your adherence to all PLOS ONE policies on sharing data and materials by including the following statement: "This does not alter our adherence to PLOS ONE policies on sharing data and materials.” (as detailed online in our guide for authors http://journals.plos.org/plosone/s/competing-interests).  If there are restrictions on sharing of data and/or materials, please state these. Please note that we cannot proceed with consideration of your article until this information has been declared.

Please include your amended Competing Interests Statement within your cover letter. We will change the online submission form on your behalf.

Please know it is PLOS ONE policy for corresponding authors to declare, on behalf of all authors, all potential competing interests for the purposes of transparency. PLOS defines a competing interest as anything that interferes with, or could reasonably be perceived as interfering with, the full and objective presentation, peer review, editorial decision-making, or publication of research or non-research articles submitted to one of the journals. Competing interests can be financial or non-financial, professional, or personal. Competing interests can arise in relationship to an organization or another person. Please follow this link to our website for more details on competing interests: http://journals.plos.org/plosone/s/competing-interests

Reply:

We have explicitly stated the funder “Euroimmun” and included the sentence “This does not alter our adherence to PLOS ONE policies on sharing data and materials” in the Competing Interests Statement. We also included this amendment within the cover letter.

# **Response to the Reviewers**:

**Reviewer #1**: The authors established a test to detect, whether CSF OCBs in patients with neuroborreliosis (NB) are specific for borrelia burgdorferi senso lato. By applying an immunoblotting test, the authors suggest that OCBs in LN are indeed specific against borrelia burgdorferi. The major limitation of this study is the limited number of patients with LN (n=10) and controls (n=11), however, the authors state in the discussion, that this is an exploratory study.

Limitations

1. What antigens for borrelia burgdorferi are available and what borrelia antigen has been used for pre-coated NCM (page 4, line 85)? The author could shortly refer to antigens (also in comparison to ELISA tests) and their specificity – also in regard to cross reactivities (maybe see Chandra A et al., 2011 Epitope mapping…).
   Reply:

Thanks for the comment. In our experiments, we used a mixture of VlsE antigens of B. afzelii (Euroimmun Charge-B: B081117RF), B. garinii (Euroimmun Charge-B: B081117RH), and B. burgdorferi sensu lato (Euroimmun Charge-B: B081117RK), as stated in the “Methods/ Isoelectric focusing followed by immunoblotting” section (page 6 line 110 – 116). To our knowledge, these are among the most relevant borrelia antigens used for immunoblots as well as for ELISA; besides that, there are also several other antigens, e.g. OspC, Flagellin, BmpA, etc.. We have added a passage to the discussion section that explains why we have chosen VlsE antigens for our experiments and addressed the issue of specificity.

2. The authors could shortly summarize common borrelia specific tests and their sensitivity / specificity (maybe as a table). For example CSF CXCL13 has been suggested as a clinical test to detect NB, although CXCL13 is not specific for NB (Rupprecht et al., 2018, Diagnostic value of CSF CXCL13…; Lepennetier et al, 2019, Cytokine and immune cell…).
   What is the advantage of the new test?
   Could the test be introduced in a clinical setting to improve diagnostics?
   Reply:

We performed the present experiments to investigate whether CSF OCB are specific to borrelia antigens in patients fulfilling the diagnostic criteria of neuroborreliosis. This study was driven by a fundamental interest in CSF research. As ELISA also uses VlsE as antigen and can be easily performed and the determination of antibody specificity index (ASI) is part of the diagnostic criteria, we do not think that antigen-specific OCB detection will be introduced in a routine setting. As IEF followed by OCB is per se a labor-intensive, costly, time-consuming and rater-dependent method, the pre-coating step is another further complicating this method.

We added a passage to the discussion section relating the diagnostic value (sensitivity and specificity) of borrelia-specific OCB to the established parameters ASI and CXCL-13.

3. Why do the authors apply a cut-off of > 2 bands for the pre-coated NCM test? Although OCBs are usually considered positive when >2 bands are specifically detected in CSF, why should this approach by applied for the immunoblotting test, especially in the context of the positive control? Please reconsider this issue.
   Reply:

Thanks for raising this important question. For the detection of “regular” OCB, no clear cut-off defining positivity is recommended by any CSF guidelines, however, most of the studies reported a cut-off of >1 or >2 CSF-restricted bands, and applying the higher cut-off results in a higher diagnostic specificity. There are several reasons for using higher cut-offs (i.e. not >0). The assessment of OCB by visual inspection is rater-dependent, and previous studies have shown that inter-rater agreement on the number of OCB, i.e. to decide to count a single band or not, is only low. Furthermore, there are also methodological issues that can hamper assessing CSF bands, e.g. non-linearity of the pH gradient used for IEF or physiological microheterogeneity of IgG. Due to these considerations, we decided to use the same cut-off (>2 bands) also for evaluation of borrelia-specific bands. We’ve added a passage that addresses this issue to the discussion section.

4. It would be interesting, if the control patient (page 9, line 181) with positive OCBs in the immunoblotting test has a positive antibody titer – at least in serum.

Reply:

We fully agree. However, as this was a control, ASI was not determined for routine procedures; and after performing our experiments, not enough sample volume was left to determine borrelia antibodies. We have stated this in the discussion section.

5. Table 1: CSF standard finding could be more detailed (e.g. Ig index, intrathecal Ig production, glucose. Lactate …)

Reply:

We have added findings of the remaining CSF routine parameters (i.e. GluR, Qalb, IgG index, intrathecal fraction of IgG, IgA and IgM, OCB) to the table.

6. Picture quality of Figure 1A right is difficult which may be due to submission format.
   Reply:

Probably yes.

7. It would have been interesting to correlate CXCL13 values (as a measure of B cell activity) with OCBs and test results.

Reply:

We fully agree. Correlations between known diagnostic tools (e.g. CXCL13, anti-borrelia specific antibody index – see also comment of Reviewer 2) and the number of borrelia-specific OCB would be interesting.

We did not find a correlation between anti-borrelia specific antibody index and the number of OCB on pre-coated NCM.

We did not measure CXCL13 (as this is not part of the routine diagnostics in our clinic).

We thank the reviewer for the careful consideration and helpful comments!

**Reviewer #2**: In this retrospective, cross-sectional case-control study CSF-serum sample pairs of 21 patients (10 with neuroborreliosis and 11 OCB positive controls) were included. The authors aimed to determine whether CSF OCBs in patients with neuroborreliosis are specific for borrelia burgdorferi. Isoelectric focusing followed by immunoblotting using (A) an uncoated as well as (B) a borrelia antigen pre-coated nitrocellulose membrane was performed. The results indicate borrelia-specific bands in 9 of 10 neuroborreliosis patients. The manuscript is well written and concise.

It might profit in view of the reviewer from additionally addressing a few minor points:

(1) Methods section: please indicate that CSF-serum sample pairs were analyzed

Reply:

Thanks for careful reading. We explicitly stated that IEF was performed in CSF and serum sample pairs. We further included OCB pattern (according to Freedman et al. Arch Neurol. 2005;62:865-870) in the text of the methods section as well as in Table 1 (NB group: 10 patients with CSF-restricted bands (pattern II), control group: 10 patients with pattern II, 1 patient with mirror bands in CSF and serum (pattern IV)).

(2) Figure 1 could be extended by another example C (OCB type 2, e.g., MS CSF-serum
    pair).

Reply:

Thanks for this comment. In Figure 1, we showed the example of a NB patient, who showed reproducible OCB on pre-coated NCM (i.e. borrelia-specific OCB), as well as the example of a control patient, who did not have reproducible OCB on pre-coated NCM (i.e. without borrelia-specific OCB). We feel that another example does not add further information.

(3) Please discuss the possible applications of this test method. Are there any
    advantages with regard to the current standard (sample volume, costs, ..)?

Reply:

We performed the present experiments to investigate whether CSF OCB are specific to borrelia antigens in patients fulfilling the diagnostic criteria of neuroborreliosis. This study was driven by a fundamental interest in CSF research. As ELISA also uses VlsE as antigen, can be easily performed, as the determination of antibody specificity index (ASI) is part of the diagnostic criteria, and as IEF followed by OCB is per se a labor-intensive, costly, time-consuming and rater-dependent method and as the pre-coating step is another further complicating this method, we do not think that antigen-specific OCB detection will be introduced in a routine setting.

(4) In table 2: Are the data normally distributed? If not, please indicate the median
    and range or IQR. Please indicate the OCB positivity rate in each group. Visual analysis
    NB uncoated 100 vs pre-coated 90%, Controls 100 vs 0%; photometric analysis NB 100 vs 90% (?), Controls 90,9% vs 0% (?).

Reply:

Thanks for the comment. We have further provided median (IQR) of the OCB number as well as the n (%) of OCB positives per group.

(5) Not sure whether the sample size is sufficient to make statements on diagnostic
    accuracy, although the results are conclusive. This could be determined in a larger study?

Reply:

We fully agree. Even though the present results suggest a high diagnostic sensitivity and specificity, further studies including a larger sample size are needed to replicate these findings. We addressed this point in the discussion section.

(6) More from academic interest than practical relevance: Is there a correlation between
    anti-borrelia specific antibody indices and the number of borrelia specific OCBs?

Reply:

Thanks for raising this interesting point. We did not find any correlation between anti-borrelia specific antibody index and the number of borrelia-specific OCB.

We thank the reviewer for the careful consideration and helpful comments!
